# A stabilized linear finite element method for anisotropic poroelastodynamics with application to cardiac perfusion

**DOI:** 10.1016/j.cma.2022.115877

**Published:** 2023-01-07

**Authors:** Namshad Thekkethil, Simone Rossi, Hao Gao, Scott I. Heath Richardson, Boyce E. Griffith, Xiaoyu Luo

**Affiliations:** a School of Mathematics and Statistics, University of Glasgow, Glasgow, UK; b Department of Mathematics, University of North Carolina, Chapel Hill, NC, USA; c Departments of Mathematics, Applied Physical Sciences, and Biomedical Engineering, University of North Carolina, Chapel Hill, NC, USA

**Keywords:** Poroelasticity, Finite element method, Variational multiscale stabilization, Perfusion, Left ventricle

## Abstract

We propose a variational multiscale method stabilization of a linear finite element method for nonlinear poroelasticity. Our approach is suitable for the implicit time integration of poroelastic formulations in which the solid skeleton is anisotropic and incompressible. A detailed numerical methodology is presented for a monolithic formulation that includes both structural dynamics and Darcy flow. Our implementation of this methodology is verified using several hyperelastic and poroelastic benchmark cases, and excellent agreement is obtained with the literature. Grid convergence studies for both anisotropic hyperelastodynamics and poroelastodynamics demonstrate that the method is second-order accurate. The capabilities of our approach are demonstrated using a model of the left ventricle (LV) of the heart derived from human imaging data. Simulations using this model indicate that the anisotropicity of the myocardium has a substantial influence on the pore pressure. Furthermore, the temporal variations of the various components of the pore pressure (hydrostatic pressure and pressure resulting from changes in the volume of the pore fluid) are correlated with the variation of the added mass and dynamics of the LV, with maximum pore pressure being obtained at peak systole. The order of magnitude and the temporal variation of the pore pressure are in good agreement with the literature.

## 1. Introduction

Cardiac perfusion, a mechanism by which blood is removed and delivered to the myocardium, plays a significant role in heart function. Cardiovascular magnetic resonance imaging is the most commonly used experimental technique for acquiring perfusion data [1,2]. Computational methods such as the finite element method (FEM) provide an alternative to medical imaging and promise to provide insight into the mechanism of perfusion. Computational methods for modelling perfusion require a poroelastodynamic framework that describes the unsteady coupled interaction between the elastodynamics of a solid and fluid dynamics within the pores of the solid. Poroelasticity is also used in other areas of biomechanics, including in models of cartilage, liver, and cornea, and it also has been widely applied in geomechanics and hydrogeology [3–5]. The complete modelling of poroelastodynamics can be considered within a fluid–structure interaction framework that resolves each pore. However, it is difficult to obtain accurate information about the pores, such as the shape and connectivity between them, and simulations of models with this level of detail are generally computationally intractable. Thus, homogenized models are commonly employed [6–10]. In these reduced models, homogenization converts the small-scale phenomenon into macroscopic quantities by considering the poroelastic medium to be a homogenized mixture of solid and fluid. In such models, the solid part is often called the skeleton [11,12]. For cardiac perfusion, the myocardium is considered a homogeneous mixture of a solid skeleton compartment consisting of cardiac myocytes and collagen and a fluid compartment composed of coronary vessels to transport blood to and from the myocardium [11–13]. In homogenization, the length scale of the pores is characterized by capillary permeability and porosity [14]. Using the poroelastic approach, the Darcy flow assumption provides a continuous flow velocity and pressure in the pores [15]. Assuming Darcy flow also simplifies the model by considering the velocity as the pressure gradient multiplied by the permeability tensor [16].

Simulating poroelastodynamics in complex problems such as cardiac perfusion requires methods that can handle the incompressibility or near incompressibility of the anisotropic, nonlinear skeleton together with Darcy flow. In large deformation models, the need to satisfy the Ladyzhenskaya-Babuska-Brezzi (LBB) criterion [17] makes it challenging to treat the skeleton as either incompressible or nearly incompressible. Costa et al. [18] were among the first to perform a complete three-dimensional finite element analysis of the left ventricle (LV) of the heart with large deformation and a nonlinear and non-homogeneous incompressible model. They concluded that higher-order finite elements are required for a stable, accurate solution. Yang et al. [19] conducted an FEM analysis of poroviscoelastic soft tissue using biquadratic interpolation for the displacement of the skeleton and fluid pressure. They concluded that the same order of interpolation for displacement and pressure would not satisfy the LBB criterion. Chapelle et al. [11] studied the poroelastodynamics in an elliptical LV with near incompressibility of the skeleton using FEM with first-order elements for the displacement and piecewise constant elements for the pressure. Richardson et al. [13] studied the poroelastodynamics in a realistic human LV using an Immersed Boundary/Finite Element method [20] that used first-order elements for both the displacement and velocity of the skeleton and the pore pressure together with stabilization using a volumetric energy term. Cookson et al. [21] used a mixed method with second-order elements for the displacement and first-order elements for the pressure for a comparably realistic LV. Lee et al. [22] used a similar approach for the analysis of poroelastodynamics in a porcine LV.

It is well known that in the absence of stabilization, using first-order elements for both the displacement and the pressure in incompressible and nearly incompressible material models leads to spurious pressure checkerboard modes and volumetric locking [23]. Prior work yielded a stable method for incompressible elasticity and poroelasticity through higher-order elements and reduced integration methods [24]. Because higher-order elements are computationally expensive, another choice is to use first-order elements for the displacement and piecewise constant elements for the pressure. However, such methods give only first-order accuracy for the pressure and are limited to near incompressibility. Obtaining second-order accuracy for both displacement and pressure requires at least linear elements for both displacement and pressure. Many stabilization techniques have been developed to enable the use of equal-order elements, such as formulations that use a modified deformation gradient [23] or mean dilation [25]. Developments in the finite element method for fluid mechanics led to a stabilized method for incompressible fluid with first-order elements for velocity and pressure [26,27]. Later, this method was extended to incompressible static elasticity [28]. Klaas et al. [29] presented the stabilized finite element method for incompressible and nearly incompressible static nonlinear hyperelasticity using linear elements for both displacement and pressure. Masud and Truster [30] used a variational multiscale framework for the stabilization using linear elements. Scovazzi et al. [31] and Zeng et al. [32] extended this method to unsteady formulations by adding a stabilization term corresponding to the inertia force using the Dynamic Variational Multiscale (D-VMS) method. Rossi et al. [33] presented a detailed formulation of the stabilized finite element method for unsteady hyperelasticity using an implicit time-stepping method.

This study extends the D-VMS method for large deformation hyperelasticity and isotropic materials presented in Rossi et al. [33] to a poroelastic anisotropic formulation suitable for complex biomechanical models, including the LV. The stabilization term in our formulation includes contributions from the anisotropic elastodynamics and poroelastodynamics. Grid convergence studies for anisotropic elastodynamics and poroelastodynamics test cases are presented. Further verification studies are reported for three-dimensional problems of shrinking and expanding cubes [11]. The method’s capabilities are demonstrated by simulating LV dynamics and perfusion using an anatomical model derived from human image data across three consecutive cardiac cycles.

## 2. Mathematical formulation

For the poroelastic medium, the homogeneous mixture formulation represents both solid and fluid as a single continuum with porosity *ϕ*, which represents the fraction of fluid volume in the porous medium, such that the volume occupied by the fluid at time *t* is

(1) $$V^{\text{f}}(t)=\int_{\Omega_{t}}\phi (x,t)\text{d}x,$$

in which *Ω_t_* is the physical region occupied by the poroelastic medium at time *t*. The volume of the skeleton is the difference between the total volume of *Ω_t_* and the fluid volume *V*^f^(*t*),

(2) $$V^{\text{s}}(t)=V(t)-V^{\text{f}}(t)=\int_{\Omega_{t}}(1-\phi (x,t))\text{d}x.$$

The deformation gradient tensor **F** of the homogeneous mixture is computed with respect to the skeleton configuration and is defined by

(3) $$F=\frac{\partial x}{\partial X},$$

in which ***X*** are material coordinates of the skeleton in the reference configuration and ***x*** = ***χ*** (***X***, *t*) are the current coordinates of the material point ***X*** at time *t*. The Lagrangian displacement and velocity of the material are ***u***(***X***, *t*) = ***x*** − ***X*** and $v(X,t)=\dot{u}(X,t)$. We consider the balance equations for the poroelastodynamics problem in Lagrangian form. Denoting the Jacobian determinant of the deformation mapping by ***J***(***X***, *t*) = det **F**, the volumes are

(4) $$V^{\text{f}}(t)=\int_{\Omega_{0}}J\phi \;(\chi (X,\;t),t)\text{d}X,\;V^{\text{f}}(0)=\int_{\Omega_{0}}\phi_{0}(X)\text{d}X,$$

(5) $$V^{\text{s}}(t)=\int_{\Omega_{0}}J(1-\phi \;(\chi \;(X,t),t))\text{d}X,\;V^{\text{s}}(0)=\int_{\Omega_{0}}\left(1-\phi_{0}(X)\right)\text{d}X,$$

in which *Ω*_0_ is the physical region occupied by the poroelastic medium in the reference configuration and *ϕ*_0_(***X***) = *ϕ* (***χ*** (***X***, 0), 0) is the porosity field in the reference configuration.

In the next section, we start by presenting the mathematical statement of the elastodynamics problem without the presence of the pore fluid (*ϕ* ≡ 0). Then, we extend the formulation to poroelastodynamics using the mass and momentum balance equations in the pore-fluid, coupled with the mass and momentum balance equations for the homogeneous mixture.

### 2.1. Elastodynamics

The dynamics of a hyperelastic solid are fully described by the equations of mass and momentum balance. Because there is no pore fluid, the homogeneous mixture is comprised solely of the skeleton and is referred to as a solid. The mass balance equation states that the mass of a solid *M*(*t*) does not change in time: *M*(*t*) = *M*(0). Introducing the mass per unit volume in the current configuration *ρ*^s^(***x***, *t*) and reference configuration $\rho_{0}^{\text{s}}(X)=\rho^{\text{s}}(\chi (X,\;0),\;0)$, we have

(6) $$M(t)=\int_{\Omega_{t}}\rho^{\text{s}}(x,t)\;\text{d}x=\int_{\Omega_{0}}J\rho^{\text{s}}(\chi (X,t),t)\;\text{d}X=\int_{\Omega_{0}}\rho_{0}^{\text{s}}(X)\;\text{d}X=M(0),$$

or, locally,

(7) $$J\rho^{\text{s}}(\chi (X,t),t)=\rho_{0}^{\text{s}}(X).$$

For an incompressible solid, *J* ≡ 1 and $\rho^{\text{s}}(\chi (X,t),t)\equiv \rho_{0}^{\text{s}}(X)$. With this, differentiating the above equation with respect to time yields the rate form, $\dot{J}=J\nabla \cdot v=0$, in which ∇ · is the divergence in the current configuration. Because ***v*** = ***v***(***X***, *t*) is the Lagrangian structural velocity, this is stated in fully Lagrangian form by

(8) $$H:\nabla_{X}v=0,$$

in which **H** = *J***F**^−*T*^ is the cofactor matrix of **F**. The cofactor matrix can be written more conveniently using the cross product between matrices [34], $H=\frac{1}{2}F\times F$(In fact, because the cross-product is a linear operator, in this form, the linearization of the cofactor is straightforward.).

Momentum balance in the Lagrangian frame is

(9) $$\rho_{0}^{\text{s}}\dot{v}=\nabla_{X}\cdot (FS)+\rho_{0}^{\text{s}}b,$$

in which ***b*** is the body force per unit mass, and **S** is the second Piola–Kirchhoff (PK2) stress. To model the myocardium, we assume the PK2 stress can be additively decomposed into active and passive parts: **S** = **S**_a_ + **S**_p_. The active stress **S**_a_ is obtained from a contraction model, whereas the passive stress **S**_p_ is obtained from a strain energy function *Ψ* = *Ψ*(**C**), such that

(10) $$S_{\text{p}}=2\frac{\partial \Psi \;(C)}{\partial C},$$

in which **C** = **F***^T^***F** is the right Cauchy–Green strain tensor. Eq. (10) corresponds to a hyperelastic material description. To enforce the constraint (8), we introduce the Lagrange multiplier *p*(***X***, *t*), which is identified as the solid pressure [22]. Next, introducing the distortional component of the deformation gradient tensor $\bar{F}=J^{-1/3}F$ and the corresponding distortional Cauchy–Green strain $\bar{C}={\bar{F}}^{T}\bar{F}$, the strain energy function is

(11) $$\Psi \;(C,p)=W\;(\bar{C})-p\;(J-1),$$

and the passive component of the PK2 stress becomes

(12) $$S_{\text{p}}=2\frac{\partial W\;(\bar{C})}{\partial C}-pJC^{-1},$$

in which $W(\bar{C})$ is the strain energy function corresponding to the isochoric deformations. Considering the Lagrange multiplier *p*(***X***, *t*) to include any spherical component of the total stress, the total PK2 stress is modified to include only the deviatoric part of the active stress, given as

(13) $$S=S_{\text{p}}+\mathrm{DEV}\left[S_{\text{a}}\right]=2\frac{\partial W\;(\bar{C})}{\partial C}-pJC^{-1}+\mathrm{DEV}\left[S_{\text{a}}\right].$$

Here, DEV[**S**] is the PK2 stress tensor corresponding to the deviatoric Cauchy stress [35], given as

(14) $$\mathrm{DEV}[S]=JF^{-1}\mathrm{dev}[\sigma ]F^{-T}=S-\frac{1}{3}(S:C)C^{-1},$$

where

(15) $$\mathrm{dev}[\sigma ]=\sigma -\frac{1}{3}tr(\sigma )I.$$

in which dev[*σ*] is the deviatoric component of the Cauchy stress *σ*. Because $\mathrm{DEV}[S]=2\partial W(\bar{C})/\partial C+\mathrm{DEV}\left[S_{\text{a}}\right]$, and the first Piola–Kirchhoff stress tensor corresponding to the deviatoric Cauchy stress $\mathrm{Dev}[P]=J\mathrm{dev}[\sigma ]F^{-T}=P-\frac{1}{3}(P:F)F^{-T}=F\;\mathrm{DEV}[S]$, the final system of equations is

(16a) $$\dot{u}=v,$$

(16b) $$\rho_{0}^{\text{s}}\dot{v}=\nabla_{X}\cdot (\mathrm{Dev}[P])-\nabla_{X}\cdot (pH)+\rho_{0}^{\text{s}}b,$$

(16c) $$0=H:\nabla_{X}v.$$

Using Eq. (16a), it is possible to write the momentum equation (Eq. (16b)) as a second-order differential equation in time. However, since a key part of the proposed stabilization method (explained later in Section 3.2) is that it acts on the velocity field, it is convenient to write the equation as a first order system. As shown in Rossi et al. [33], after discretization, this choice results in a two-step algorithm in which we first solve for the velocity and pressure fields and then update the displacement field. Note that, as demonstrated in Scovazzi et al. [31] for isotropic incompressible and nearly incompressible elasticity, we obtain a stable scheme for the dynamic problem only with velocity stabilization.

### 2.2. Poroelastodynamics

Although we consider both the skeleton and the pore fluid as incompressible, the homogeneous mixture may not be incompressible due to the fluid moving within the skeleton [11,36]. The total mass of the homogeneous mixture as a function of time is *M*(*t*) = *M*^f^(*t*) + *M*^s^(*t*) and is evaluated via

(17) $$M^{\text{f}}(t)=\int_{\Omega_{t}}\phi (x,t)\rho^{\text{f}}(x,t)\;\text{d}x,\;M^{\text{s}}(t)=\int_{\Omega_{t}}(1-\phi (x,t))\rho^{\text{s}}(x,t)\;\text{d}x.$$

The density of the mixture is *ρ*(***x***, *t*) = *ϕ*(***x***, *t*)*ρ*^f^(***x***, *t*) + (1 − *ϕ*(***x***, *t*))*ρ*^s^(***x***, *t*). For an unconfined poroelastic medium, even if the fluid and the solid are incompressible, the mixture is generally not incompressible (*ρ* ≠ *ρ*_0_) due to the presence of an external source. For cardiac perfusion, the porous medium (myocardium) is connected to an external source (large blood vessels) and is unconfined. Mass conservation for the solid skeleton means *M*^s^(*t*) ≡ *M*^s^(0), and the change of mass of the mixture at time *t* is *ΔM* = *M*(*t*) − *M*(0) = *M*^f^(*t*) − *M*^f^(0). From (17), the local form of the mass balance equations for the solid and fluid compartments in the reference configuration become

(18a) $$J\rho^{\text{s}}(\chi (X,t),t)(1-\phi (\chi (X,t),t))=\rho_{0}^{\text{s}}(X)\left(1-\phi_{0}(X)\right),$$

(18b) $$J\rho^{\text{f}}(\chi (X,t),t)\phi (\chi (X,t),t)-\rho_{0}^{\text{f}}(X)\phi_{0}(X)=m(X,t),$$

in which *m*(***X***, *t*) is the (Lagrangian) added mass, i.e., the additional fluid mass per unit reference volume. For an incompressible solid, $\rho^{\text{s}}\equiv \rho_{0}^{\text{s}}$. Taking the time derivative of Eq. (18b), and using $\dot{J}=J\nabla \cdot v$, we find $J\rho^{\text{f}}\phi (\nabla \cdot v)+J\left(\rho^{\text{f}}\phi \right)=\dot{m}$. This can be simplified as

(19) $$\frac{\partial \left(\rho^{\text{f}}\phi \right)}{\partial t}+\nabla \cdot \left(\rho^{\text{f}}\phi v\right)=\frac{\dot{m}}{J}$$

Using the definition of perfusion velocity ***w***(***x***, *t*) = *ϕ*(***x***, *t*)(***v***^f^(***χ***^−1^(***x***, *t*), *t*) − ***v***(***χ***^−1^(***x***, *t*), *t*)), and defining the Eulerian mass source

(20) $$\rho^{\text{f}}s=\frac{\partial \left(\rho^{\text{f}}\phi \right)}{\partial t}+\nabla \cdot \left(\rho^{\text{f}}\phi v^{\text{f}}\right)$$

in which *s*(***x***, *t*) is the volumetric source in the current configuration, we obtain, after algebraic manipulation,

(21) $$\frac{\dot{m}}{J}+\nabla \cdot \left(\rho^{\text{f}}w\right)=\rho^{\text{f}}s.$$

In the formulation used herein, the perfusion velocity is assumed to be governed by a Darcy flow model, such that

(22) $$w(x,t)=-K\nabla p^{\text{pore}}\left(\chi^{-1}(x,t),t\right),$$

in which *p*^pore^(***X***, *t*) is the pore pressure in the reference configuration and **K** is the permeability tensor. We assume the total pore pressure *p*^pore^(***X***, *t*) is the sum of three contributions: the Lagrange multiplier *p*(***X***, *t*) that impose skeleton incompressibility; the pressure derived from the pore pressure–volume relationship *p*^PV^(***X***, *t*); and the compaction pressure *p*^c^(***X***, *t*) that enforces a non-negative porosity *ϕ* at all times, so that

(23) $$p^{\text{pore}}(X,t)=p(X,t)+p^{\text{PV}}(X,t)+p^{\text{c}}(X,t),$$

in which *p*^PV^ and *p*^c^ are functions (specified later) of the added mass *m*. Because the pore pressure and the added mass are defined in the reference configuration, Eq. (21) is pulled back to the reference configuration using the incompressibility assumption for the fluid, $\rho^{\text{f}}=\rho_{0}^{\text{f}}$, along with Eqs. (22) and (23) to obtain the Lagrangian form of the added mass equation,

(24) $$\frac{1}{\rho_{0}^{\text{f}}}\dot{m}-\nabla_{X}\cdot \left(K_{0}\nabla_{X}p\right)-\nabla_{X}\cdot \left(K_{0}^{m}\nabla_{X}m\right)=S,$$

in which the Lagrangian permeability tensor is **K**_0_ = *J***F**^−1^**KF**^−*T*^, the added mass dependent permeability tensor is $K_{0}^{m}=K_{0}\partial \left(p^{\text{PV}}+p^{\text{c}}\right)/\partial m$, and the Lagrangian volumetric source is *S*(***X***, *t*).

The constraint that the skeleton remain incompressible, *J*(1 − *ϕ*) = 1 − *ϕ*_0_, can be written in terms of the added mass *m*. In fact, by using Eq. (18) to eliminate *ϕ*, and using the fluid incompressibility assumption $\rho^{\text{f}}=\rho_{0}^{\text{f}}$, we find the relationship $J-1=m/\rho_{0}^{\text{f}}$. Then, the incompressibility constraint in Lagrangian coordinates can be stated in rate form as

(25) $$H:\nabla_{X}v=\frac{\dot{m}}{\rho_{0}^{\text{f}}}.$$

Momentum balance of the mixture involves the inertia of both the skeleton and the pore fluid. Assuming the fluid and the skeleton have the same acceleration, we can simplify the equations of momentum balance, obtaining

(26) $$\left(\rho_{0}+m\right)\dot{v}=\nabla_{X}\cdot (\mathrm{Dev}[P])-\nabla_{X}\cdot (pH)+\rho_{0}b,$$

in which $\rho_{0}=\rho (\chi (X,0),0)=\rho_{0}^{\text{f}}\phi_{0}+\rho_{0}^{\text{s}}\left(1-\phi_{0}\right)$

The stress tensor **S** and the fluid pressures *p*^PV^ and *p*^c^ are derived from an energy function that depends on the strain tensor $\bar{C}$, the Lagrange multiplier *p* and the added mass *m*, such that $\Psi =\Psi (\bar{C},p,m)$.

(27) $$\Psi (C,p,m)=W(\bar{C})+U(m)-p\left(J-1-\frac{m}{\rho_{0}^{\text{f}}}\right),$$

in which $W(\bar{C})$ characterizes the skeleton isochoric deformations, *U*(*m*) characterizes the changes in volume due to the fluid motion, and the last term enforces the incompressibility constraint of the solid skeleton. The above strain energy function allows for negative values of *ϕ* depending on the value of the source *S*. To enforce a non-negative value for the porosity *ϕ*, we split the energy *U*(*m*) into a pressure–volume relation *U*^PV^(*m*) and a compaction penalization *U*^c^(*m*), such that

(28) $$\Psi (C,p,m)=W(\bar{C})+U^{\text{PV}}(m)+U^{\text{c}}(m)-p\left(J-1-\frac{m}{\rho_{0}^{\text{f}}}\right).$$

With this definition of the energy, we obtain

(29) $$S=2\frac{\partial W\;(\bar{C})}{\partial C}-pJC^{-1}+\mathrm{DEV}\left[S_{a}\right],\;p^{\text{PV}}=\frac{\partial U^{\text{PV}}(m)}{\partial m},\;p^{\text{c}}=\frac{\partial U^{\text{c}}(m)}{\partial m}.$$

The final system of equations for the poroelastic mixture is

(30a) $$\dot{u}=v$$

(30b) $$\left(\rho_{0}+m\right)\dot{v}=\nabla_{X}\cdot (\mathrm{Dev}[P])-\nabla_{X}\cdot (pH)+\rho_{0}b,$$

(30c) $$H:\nabla_{X}v=\frac{1}{\rho_{0}^{\text{f}}}\dot{m},$$

$$\frac{1}{\rho_{0}^{f}}\dot{m}=\nabla_{X}\cdot \left(K_{0}\nabla_{X}p\right)+\nabla_{X}\cdot \left(K_{0}^{m}\nabla_{X}m\right)+S.$$

Note that similar to Eq. (16), because the stabilization (Section 3.3) acts on the velocity field, it is convenient to write the balance equations as a first-order system.

#### Remark.

The density of the fluid *ρ*^f^ and the pore pressure *p*^pore^ are related by the free enthalpy of the fluid *g*^m^ [11], given as

(31) $$\frac{1}{\rho^{\text{f}}}=\frac{\partial g^{\text{m}}}{\partial p^{\text{pore}}}.$$

Following an isothermal assumption, this relationship is simplified as [37]

(32) $$g^{\text{m}}=\frac{p^{\text{pore}}\;-\;p_{0}^{\text{pore}}}{\rho_{0}^{\text{f}}},$$

in which $p_{0}^{\text{pore}}$ is the pore pressure in the initial reference configuration. The above choice holds if the fluid is incompressible, i.e.$\rho^{\text{f}}=\rho_{0}^{\text{f}}$.

### 2.3. Constitutive equations

We verify that our formulation is stable and robust using several constitutive laws. For isotropic elastic solids, we use the neo-Hookean and Mooney–Rivlin models,

(33) $$\;\text{Neo-Hookean:}\;W\;(\bar{C})=\frac{G}{2}\left({\bar{I}}_{1}-3\right)\text{,}$$

(34) $$\text{Mooney}-\text{Rivlin:}\;W(\bar{C})=C_{1}\left({\bar{I}}_{1}-3\right)+C_{2}\left({\bar{I}}_{2}-3\right),$$

in which *G*, *C*_1_, and *C*_2_ are material constants, and ${\bar{I}}_{1}=\mathrm{tr}(\bar{C})$ and ${\bar{I}}_{2}=\frac{1}{2}\left({\bar{I}}_{1}^{2}+\mathrm{tr}\left({\bar{C}}^{2}\right)\right)$ are invariants of $\bar{C}$. For anisotropic elastic solids, we use the standard reinforced model,

(35) $$W(\bar{C})=\frac{G}{2}\left({\bar{I}}_{1}-3\right)+\frac{G_{\text{f}}}{2}{\left(\max\left({\bar{I}}_{4\text{f}},1\right)-1\right)}^{2},$$

in which *G*_f_ is a material constant and ${\bar{I}}_{4\text{f}}=f_{0}\cdot \bar{C}f_{0}$, with ***f***_0_ being the fibre direction in the initial reference configuration.

For the pore-fluid, the energy corresponding to the pressure–volume relationship *U*^PV^(*m*) and the compaction penalization *U*^c^(*m*) are given as

(36) $$U^{\text{PV}}(m)=\frac{\kappa_{\text{s}}}{2}m^{2},\;U^{\text{c}}(m)=c\;\tan^{-1}\left(\frac{m\;+\;\phi_{0}\;-\;\phi_{\text{crit}}}{\epsilon }\right),$$

in which *κ*_s_ and *c* are constants, *ϕ*_crit_ is the critical value of *ϕ* up to which compaction is permitted, and *ϵ* is a small value. Here, *c* is the maximum value of the pore pressure from the previous time step. When (*m* + *ϕ*_0_) ≈ *ϕ*_crit_, the pore pressure component *p*^c^ given as

(37) $$p^{\text{c}}=\frac{\partial U^{\text{c}}}{\partial m}=c\frac{\epsilon^{2}}{\epsilon^{2}\;+\;{\left(m\;+\;\phi_{0}\;-\;\phi_{\text{crit}}\right)}^{2}}$$

is very large to prevent further pore fluid extraction [11,22]. The functional form of *p^c^* is chosen this way to prevent *m* + *ϕ*_0_ becoming smaller than *ϕ*_crit_. In fact, for very small value of *ϵ* ≈ 0, the contribution introduced by *p^c^* is negligible if *m* + *ϕ*_0_ ≫ *ϕ*_crit_ but becomes rapidly large as *m* + *ϕ*_0_ approaches *ϕ*_crit_. However, very small values of *ϵ* will lead to divergence of the nonlinear solver. Preliminary numerical experiments not reported here suggest that the choice *ϵ* = 0.001 and *ϕ*_crit_ = 0.001 ensures convergence of the nonlinear solver while enforcing the constraint *m* + *ϕ*0 *> ϕ*crit.

To model the biomechanics of the left ventricle, we use the Holzapfel–Ogden (HO) model [38],

(38) $$\begin{array}{l} W(\bar{C})=\frac{a}{2b}\exp\left[b\left({\bar{I}}_{1}-3\right)\right]\\ \;+\sum_{\text{i}=\text{f},\text{s}}\frac{a_{\text{i}}}{2b_{\text{i}}}\left\{\exp\left[b_{\text{i}}{\left(\max\left({\bar{I}}_{4\text{i}},1\right)-1\right)}^{2}\right]-1\right\}+\frac{a_{\text{fs}}}{2b_{\text{fs}}}\left\{\exp\left[b_{\text{fs}}{\left({\bar{I}}_{8\text{fs}}\right)}^{2}\right]-1\right\}, \end{array}$$

in which *a*, *b*, *a*_i_, *b*_i_, *a*_fs_, and *b*_fs_ are material constants, ${\bar{I}}_{4\;\text{s}}=s_{0}\cdot \bar{C}s_{0}$, and ${\bar{I}}_{8\text{fs}}=f_{0}\cdot \bar{C}s_{0}$, with ***s***_0_ the sheet direction in the reference configuration. For the pressure–volume relationship within the pores, we use the energy function proposed by Bruinsma et al. [39],

(39) $$\begin{array}{l} U^{\text{PV}}(m)=\frac{q_{1}}{q_{3}}\exp\left(q_{3}\left(m+\phi_{0}\right)\right)+q_{2}\left(m+\phi_{0}\right)\left[\ln\left(q_{3}\left(m+\phi_{0}\right)\right)-1\right]\\ \;-\left[q_{1}\exp\left(q_{3}\phi_{0}\right)+q_{2}\ln\left(\phi_{0}\right)\right]\left(m+\phi_{0}\right), \end{array}$$

in which *q*_1_, *q*_2_, and *q*_3_ are material constants.

Finally, the active component of the stress tensor is

(40) $$S_{\text{a}}=T\left(t,I_{4\text{f}}\right)f_{0}⊗f_{0},$$

in which the scalar tension *T* = *T*(*t*, *I*_4f_) is a function of time and the current fibre elongation ***I***_4f_ = ***f***_0_ · **C*f***_0_. Specifically,

(41) $$T\left(t,I_{4\text{f}}\right)=T_{\text{a}}(t)\left[1+4.9\left(\sqrt{I_{4\text{f}}}-1\right)\right],$$

in which *T_a_*(*t*) is the active tension that is determined empirically.

## 3. Numerical formulation

This section introduces a finite element method specialized to piecewise linear (*P*^1^) finite elements for the incompressible poroelastic mixture. In particular, we extend a previously developed stabilization method for *P*^1^ elements for isotropic elastodynamics [33] to anisotropic hyperelastic materials and anisotropic poroelastic materials. We use the second-order backward differentiation (BDF2) method to integrate the elastodynamic and poroelastodynamic equations in time. As in prior work [33], the BDF2 method with a time step size controlled by the CFL condition leads to accurate dynamics and dissipation of only the undesired high frequencies.

We denote by

(42) $${\left(v,w\right)}_{\Omega_{0}}=\int_{\Omega_{0}}vw\;\text{d}X\;\;\text{and}\;\;{\left(v,w\right)}_{\Omega_{0}}=\int_{\Omega_{0}}v\cdot w\text{d}X$$

the *L*^2^(*Ω*_0_) and *L*^2^(*Ω*_0_)*^d^* inner products on the interior of the domain *Ω*_0_ and by

(43) $${\langle v,w\rangle }_{\Gamma_{0}}=\int_{\Gamma_{0}}vw\;\text{d}A$$

a functional on a regular subset *Γ*_0_ of the boundary ∂*Ω*_0_. We also define the element-wise inner products

(44) $${\left(v,w\right)}_{\Omega_{0}^{\prime }}=\sum_{K\in {𝒯}^{h}}\int_{K}vw\;\text{d}X\;\text{and}\;{\left(v,w\right)}_{\Omega_{0}^{\prime }}=\sum_{K\in {𝒯}^{h}}\int_{K}v\cdot w\text{d}X\text{,}$$

in which *K* is an element of the triangulation ${𝒯}^{h}$, such that $\bar{\Omega }=\cup_{K\in {𝒯}^{h}}K$. Here we consider *K* to represent either a triangle (in two spatial dimensions) or a tetrahedron (in three dimensions). To simplify the notation, from this point on, we omit the subscript ***X*** if the inner products are taken over *Ω*_0_ or $\Omega_{0}^{\prime }$, because all quantities are Lagrangian quantities described using reference coordinates.

### 3.1. Abstract variational multiscale framework for nonlinear dynamics

We present here the abstract framework for nonlinear problems [33] using the D-VMS method. Denote by *V* an infinite dimensional space, V* its dual, and 𝒩 a nonlinear operator associated with a generic nonlinear problem whose variational statement can read as *Find* ***y*** ∈ *V such that*, ∀***_φ_*** ∈ *V*,

(45) $${\langle 𝒩\;(y),\varphi \rangle }_{V^{*},V}={\langle f,\varphi \rangle }_{V^{*},V},$$

in which ***y*** is the solution vector and $𝒩(y)\in V^{*}$. The variational multiscale framework introduces a decomposition of the space *V* into a finite dimensional subspace $\bar{V}$ and its compliment *V*′ such that $V=\bar{V}+V^{\prime }$. With this, we can now decompose into a resolvable coarse scale and unresolvable fine scale vectors $y=\bar{y}+y^{\prime }$ and $\varphi =\bar{\varphi }+\varphi^{\prime }$ such that (45) can be recast as *Find* $\bar{y}\in \bar{V}$ *and* ***y***′ ∈ *V*′ *such that*, $\forall \bar{\varphi }\in \bar{V}$ *and* ∀***φ***′ ∈ *V*′,

(46a) $${\langle 𝒩\left(\bar{y}\;+\;y^{\prime }\right),\bar{\varphi }\rangle }_{V^{*},V}={\langle f,\bar{\varphi }\rangle }_{V^{*},V},$$

(46b) $${\langle 𝒩\left(\bar{y}\;+\;y^{\prime }\right),\varphi^{\prime }\rangle }_{V^{*},V}={\langle f,\varphi^{\prime }\rangle }_{V^{*},V}.$$

Assuming the existence of the Fréchet derivative $ℒ[\bar{y}](\cdot )$ of 𝒩(⋅), as in Rossi et al. [33], defined as

(47) $$\underset{\left\|y^{\prime }\right\|\to 0}{\lim}\frac{1}{\left\|y^{\prime }\right\|}\left\|𝒩\left(\bar{y}+y^{\prime }\right)-𝒩(\bar{y})-ℒ[\bar{y}]\left(y^{\prime }\right)\right\|=0,$$

we can approximate the nonlinear problem as follows *Find* $\bar{y}\in \bar{V}$ *and* ***y***′ ∈ *V*′ *such that*, $\forall \bar{\varphi }\in \bar{V}$ *and* ∀***φ***′ ∈ *V*′,

(48a) $${\langle 𝒩(\bar{y}),\bar{\varphi }\rangle }_{V^{*},V}+{\langle ℒ[\bar{y}]\left(y^{\prime }\right),\bar{\varphi }\rangle }_{V^{*},V}={\langle f,\bar{\varphi }\rangle }_{V^{*},V},$$

(48b) $${\langle 𝒩(\bar{y}),\varphi^{\prime }\rangle }_{V^{*},V}+{\langle ℒ[\bar{y}]\left(y^{\prime }\right),\varphi^{\prime }\rangle }_{V^{*},V}={\langle f,\varphi^{\prime }\rangle }_{V^{*},V}.$$

As we will demonstrate in the numerical test, the linearized problem above results in a second-order accurate method when using linear finite elements.

Introducing the formal adjoint of ${ℒ}^{*}[\bar{y}]$ of $ℒ[\bar{y}]$, the above problem is recast as *Find* $\bar{y}\in \bar{V}$ *and* ***y***′ ∈ *V*′ *such that*, $\forall \bar{\varphi }\in \bar{V}$ *and* ∀***φ***′ ∈ *V*′,

(49a) $${\langle 𝒩(\bar{y}),\bar{\varphi }\rangle }_{V^{*},V}+{\langle y^{\prime },{ℒ}^{*}[\bar{y}]\bar{\varphi }\rangle }_{V^{*},V}={\langle f,\bar{\varphi }\rangle }_{V^{*},V},$$

(49b) $$\;{\langle ℒ[\bar{y}]y^{\prime },\varphi^{\prime }\rangle }_{V^{*},V}={\langle f-𝒩\;(\bar{y}),\varphi^{\prime }\rangle }_{V^{*},V}.$$

The solution to (49b) can be formally expressed in terms of the fine-scale Green’s function integral operator ${ℳ}^{\prime }$

(50) $$y^{\prime }={ℳ}^{\prime }(𝒩(\bar{y})-f)=-\int_{\Omega_{0}}g^{\prime }(X,Y)(𝒩(\bar{y})-f)\text{d}X\;\text{d}Y,$$

in which *g*′(·, ·) is the fine-scale Green’s function. Approximating the Green’s function using localization arguments as *g*′(***X***, ***Y***) ≈ ***τ_y_****δ*(***X*** − ***Y***), in which *δ* is the multidimensional Dirac delta function, we obtain the multiscale variational formulation, *Find* $\bar{y}\in \bar{V}$ *such that*, $\forall \bar{\varphi }\in \bar{V}$,

(51) $${\langle 𝒩(\bar{y}),\bar{\varphi }\rangle }_{V^{*},V}-{\langle \tau_{y}(𝒩\;(\bar{y})-f),{ℒ}^{*}[\bar{y}]\bar{\varphi }\rangle }_{V^{*},V}={\langle f,\bar{\varphi }\rangle }_{V^{*},V}.$$

The matrix ***τ_y_*** contains the problem dependent parameters that we use to stabilize the variational formulation.

### 3.2. Variational multiscale method for hyperelastodynamics

We now apply the variational multiscale framework (51) to the hyperelastodynamic problem (16). Using the vectors ***y*** = [***u***, ***v***, *p*] and $\varphi =[\tilde{w},w,q]$, we have

(52a) $$\begin{array}{l} {\langle 𝒩\left(y\right),\varphi \rangle }_{V^{*},V}={\left(\dot{u}-v,\tilde{w}\right)}_{\Omega_{0}}\\ \;+{\left(\rho_{0}^{\text{s}}\dot{v}-\nabla \cdot (\mathrm{Dev}[P])+\nabla \cdot (pH),w\right)}_{\Omega_{0}}+{\left(H:\nabla v,q\right)}_{\Omega_{0}}, \end{array}$$

(52b) $${\langle f,\varphi \rangle }_{V^{*},V}={\left(\rho_{0}^{\text{s}}b,w\right)}_{\Omega_{0}}.$$

Introducing the multiscale decomposition and imposing $\dot{\bar{u}}=\bar{v}$, we find that ${\dot{u}}^{\prime }=v^{\prime }$, and

(53) $$\begin{array}{l} {\langle ℒ[\bar{y}]y^{\prime },\bar{\varphi }\rangle }_{V^{*},V}={\left(\rho_{0}^{\text{s}}{\dot{v}}^{\prime }-\nabla \cdot \left[\frac{\partial \mathrm{Dev}[\bar{P}]}{\partial F}:\nabla u^{\prime }\right]+\nabla \cdot \left(\bar{p}\bar{F}\times \nabla u^{\prime }\right)+\nabla \cdot \left(p^{\prime }\bar{H}\right),\bar{w}\right)}_{\Omega_{0}^{\prime }}\\ \;+{\left(\bar{H}:\nabla v^{\prime }+\left(\bar{F}\times \nabla u^{\prime }\right):\nabla \bar{v},\bar{q}\right)}_{\Omega_{0}^{\prime }}, \end{array}$$

in which $\bar{F}$, $\bar{H}$ and $\bar{P}$ are, respectively, the deformation gradient tensor, its cofactor, and the first Piola–Kirchhoff stress tensors evaluated using the coarse scale displacements $\bar{u}$. Integration by parts leads to

(54) $$\begin{array}{l} {\langle y^{\prime },{ℒ}^{*}[\bar{y}]\bar{\varphi }\rangle }_{V^{*},V}={\left(\rho_{0}^{\text{s}}v^{\prime },\bar{w}\right)}_{\Omega_{0}^{\prime }}-{\left(u^{\prime },\nabla \cdot \left[\nabla \bar{w}:\frac{\partial \mathrm{Dev}[\bar{P}]}{\partial F}\right]\right)}_{\Omega_{0}^{\prime }}\\ \;+{\left(u^{\prime },\nabla \cdot (\bar{p}\bar{F}\times \nabla \bar{w})\right)}_{\Omega_{0}^{\prime }}+{\left(p^{\prime },\bar{H}:\nabla \bar{w}\right)}_{\Omega_{0}^{\prime }}\\ \;-{\left(v^{\prime },\bar{H}\nabla \bar{q}\right)}_{\Omega_{0}^{\prime }}-{\left(u^{\prime },(\bar{F}\times \nabla \bar{v})\nabla \bar{q}\right)}_{\Omega_{0}^{\prime }}. \end{array}$$

Using a minimalist approach to avoid spurious pressure oscillations [33], we approximate

(55) $${\langle ℒ[\bar{y}]y^{\prime },\bar{\varphi }\rangle }_{V^{*},V}\approx {\left(\bar{H}:\nabla v^{\prime },\bar{q}\right)}_{\Omega_{0}^{\prime }},$$

(56) $${\langle y^{\prime },{ℒ}^{*}[\bar{y}]\bar{\varphi }\rangle }_{V^{*},V}\approx -{\left(v^{\prime },\bar{H}\nabla \bar{q}\right)}_{\Omega_{0}^{\prime }}.$$

This approximation is equivalent to choosing

(57) $$\tau_{y}=\left[\begin{array}{lll} 0 & 0 & 0\\ 0 & 0 & 0\\ 0 & \tau & 0 \end{array}\right].$$

Introducing the finite dimensional test function spaces

(58a) $${𝒱}_{v}^{h}=\left\{w^{h}\in \left(C^{0}{\left(\Omega_{0}\right)}^{n_{d}}\right):{w^{h}|}_{K}\in {\left({𝒫}^{1}(K)\right)}^{n_{d}},\forall K\in {𝒯}^{h},w^{h}=0\;\text{on}\;\Gamma_{\text{D}}\right\}\;\text{and}$$

(58b) $${𝒱}_{p}^{h}=\left\{q^{h}\in C^{0}\Omega_{0}:{q^{h}|}_{K}\in P^{1}(K),\forall K\in {𝒯}^{h}\right\},$$

and the corresponding trial function spaces

(59a) $${𝒮}_{v}^{h}=\left\{w^{h}\in \left(C^{0}{\left(\Omega_{0}\right)}^{n_{d}}\right):{w^{h}|}_{K}\in {\left(P^{1}(K)\right)}^{n_{d}},\forall K\in {𝒯}^{h},w^{h}=v_{\text{D}}\;\text{on}\;\Gamma_{\text{D}}\right\}\;\text{and}$$

(59b) $${𝒮}_{p}^{h}={𝒱}_{p}^{h},$$

the stabilized weak form of the hyperelastodynamic equations reads *Find* ***u****^h^*, $v^{h}\in {𝒮}_{v}^{h}$, *and* $p^{h}\in {𝒮}_{q}^{h}$, *such that*, $\forall {\tilde{w}}^{h}$, $w^{h}\in {𝒱}_{v}^{h}$, *and* $q^{h}\in {𝒱}_{q}^{h}$,

(60a) $$0={\left({\dot{u}}^{h}-v^{h},{\tilde{w}}^{h}\right)}_{\Omega_{0}},$$

(60b) $$0={\left(\rho_{0}^{\text{s}}{\dot{v}}^{h},w^{h}\right)}_{\Omega_{0}}+{\left(\mathrm{Dev}\left[P^{h}\right]-p^{h}H^{h},\nabla w^{h}\right)}_{\Omega_{0}}-{\langle t_{0},w^{h}\rangle }_{\Gamma_{\text{N}}}-{\left(\rho_{0}^{\text{s}}b,w^{h}\right)}_{\Omega_{0}},$$

(60c) $$0={\left(H^{h}:\nabla v^{h},q^{h}\right)}_{\Omega_{0}}+{\left(v^{\prime },H^{h}\nabla q^{h}\right)}_{\Omega_{0}},$$

(60d) $$v^{\prime }=-\tau \left({\dot{v}}^{h}+\frac{1}{\rho_{0}^{s}}H^{h}\nabla p^{h}-b\right),$$

in which the terms corresponding to the deviatoric component of the stress tensor disappear because of the choice of linear shape functions. We use the BDF2 scheme for the time discretization and Newton’s method for the nonlinear equations. After discretization, as shown in Rossi et al. [33], the system can be solved as a two-step algorithm in which we first solve for the velocity and pressure fields, followed by an explicit update of the displacement field. The numerical implementation follows that given by Rossi et al. [33].

### 3.3. Variational multiscale method for poroelastodynamics

We now apply the variational multiscale framework (51) to the poroelastodynamic problem (30). Using the vectors *y* = [***u***, ***v***, *p*, *m*] and $\varphi =[\tilde{w},w,q,r]$, we have

(61a) $$\begin{array}{l} {\langle 𝒩\left(y\right),\varphi \rangle }_{V^{*},V}={\left(\dot{u}-v,\tilde{w}\right)}_{\Omega_{0}}\\ \;+{\left(\left(\rho_{0}+m\right)\dot{v}-\nabla \cdot (\mathrm{Dev}[P])+\nabla \cdot (pH),w\right)}_{\Omega_{0}}\\ \;+{\left(H:\nabla v-\frac{\dot{m}}{\rho_{0}^{\text{f}}},q\right)}_{\Omega_{0}}\\ \;+{\left(\frac{1}{\rho_{0}^{\text{f}}}\dot{m}-\nabla \cdot \left(K_{0}\nabla p\right)-\nabla \cdot \left(K_{0}^{m}\nabla m\right),r\right)}_{\Omega_{0}}, \end{array}$$

(61b) $${\langle f,\varphi \rangle }_{V^{*},V}={\left(\rho_{0}b,w\right)}_{\Omega_{0}}+{\left(S,r\right)}_{\Omega_{0}},$$

Introducing the multiscale decomposition as before, we find

(62) $$\begin{array}{l} {\langle ℒ[\bar{y}]y^{\prime },\bar{\varphi }\rangle }_{V^{*},V}={\left(m^{\prime }\dot{\bar{v}}+\left(\rho_{0}+\bar{m}\right){\dot{v}}^{\prime }-\nabla \cdot \left[\frac{\partial \mathrm{Dev}[\bar{P}]}{\partial F}:\nabla u^{\prime }\right]+\nabla \cdot \left(\bar{p}\bar{F}\times \nabla u^{\prime }\right)+\nabla \cdot \left(p^{\prime }\bar{H}\right),\bar{w}\right)}_{\Omega_{0}^{\prime }}\\ \;+{\left(\bar{H}:\nabla v^{\prime }+\left(\bar{F}\times \nabla u^{\prime }\right):\nabla \bar{v}-\frac{{\dot{m}}^{\prime }}{\rho_{0}^{\text{f}}},\bar{q}\right)}_{\Omega_{0}^{\prime }}\\ \;+{\left(\frac{1}{\rho_{0}^{\text{f}}}{\dot{m}}^{\prime }-\nabla \cdot \left({\bar{K}}_{0}\nabla p^{\prime }\right)-\nabla \cdot \left({\bar{K}}_{0}^{m}\nabla m^{\prime }+\left(\frac{\partial {\bar{K}}_{0}^{m}}{\partial m}m^{\prime }\right)\nabla \bar{m}\right),\bar{r}\right)}_{\Omega_{0}^{\prime }}, \end{array}$$

in which ${\bar{K}}_{0}=\bar{J}{\bar{F}}^{-1}K{\bar{F}}^{-T}$ and ${\bar{K}}_{0}^{m}={\bar{K}}_{0}\partial \left({\bar{p}}^{\text{PV}}+{\bar{p}}^{\text{c}}\right)/\partial \bar{m}$. We remark that in (62), we consider the permeability tensor **K**_0_ to be a model parameter defined in the reference configuration. If **K**_0_ is defined in current configuration, then Eq. (62) should contain its linearization in terms of the displacements. Integration by parts leads to

(63) $$\begin{array}{l} {\langle y^{\prime },{ℒ}^{*}[\bar{y}]\bar{\varphi }\rangle }_{V^{*},V}={\left(m^{\prime }\dot{\bar{v}},\bar{w}\right)}_{\Omega_{0}^{\prime }}+{\left(\rho_{0}^{\text{s}}{\dot{v}}^{\prime },\bar{w}\right)}_{\Omega_{0}^{\prime }}-{\left(u^{\prime },\nabla \cdot \left[\nabla \bar{w}:\frac{\partial \mathrm{Dev}[\bar{P}]}{\partial F}\right]\right)}_{\Omega_{0}^{\prime }}\\ \;+{\left(u^{\prime },\nabla \cdot (\bar{p}\bar{F}\times \nabla \bar{w})\right)}_{\Omega_{0}^{\prime }}+{\left(p^{\prime },\bar{H}:\nabla \bar{w}\right)}_{\Omega_{0}^{\prime }}\\ \;-{\left(v^{\prime },\bar{H}\nabla \bar{q}\right)}_{\Omega_{0}^{\prime }}-{\left(u^{\prime },(\bar{F}\times \nabla \bar{v})\nabla \bar{q}\right)}_{\Omega_{0}^{\prime }}+{\left(\frac{1}{\rho_{0}^{\text{f}}}{\dot{m}}^{\prime },\bar{q}\right)}_{\Omega_{0}^{\prime }}\\ \;+{\left(\frac{1}{\rho_{0}^{\text{f}}}{\dot{m}}^{\prime },\bar{r}\right)}_{\Omega_{0}^{\prime }}-{\left(p^{\prime },\nabla \cdot \left({\bar{K}}_{0}\nabla \bar{r}\right)\right)}_{\Omega_{0}^{\prime }}+-{\left(p^{\prime },\nabla \cdot \left({\bar{K}}_{0}\nabla \bar{r}\right)\right)}_{\Omega_{0}^{\prime }}, \end{array}$$

in which we assume ${\bar{K}}_{0}={\bar{K}}_{0}^{T}$. Using again a minimalist approach to avoid spurious pressure oscillations, we choose

(64) $$\tau_{y}=\left[\begin{array}{llll} 0 & 0 & 0 & 0\\ 0 & 0 & 0 & 0\\ 0 & \tau & 0 & 0\\ 0 & 0 & 0 & 0 \end{array}\right].$$

Using the function spaces defined in (58) and (59), the stabilized weak form of the poroelastodynamic equations reads, *Find* ***u****^h^*, $v^{h}\in {𝒮}_{v}^{h}$, *and p^h^*, $m^{h}\in {𝒮}_{q}^{h}$, *such that*, $\forall {\tilde{w}}^{h}$, $w^{h}\in {𝒱}_{v}^{h}$, *and q^h^*, $r^{h}\in {𝒱}_{q}^{h}$,

(65a) $$0={\left({\dot{u}}^{h}-v^{h},{\tilde{w}}^{h}\right)}_{\Omega_{0}},$$

(65b) $$0={\left(\left(\rho_{0}+m^{h}\right){\dot{v}}^{h},w^{h}\right)}_{\Omega_{0}}+{\left(\mathrm{Dev}\left[P^{h}\right]-p^{h}H^{h},\nabla w^{h}\right)}_{\Omega_{0}}-{\langle t_{0},w^{h}\rangle }_{\Gamma_{\text{N}}}-{\left(\rho_{0}^{\text{s}}b,w^{h}\right)}_{\Omega_{0}},$$

(65c) $$0={\left(H^{h}:\nabla v^{h},q^{h}\right)}_{\Omega_{0}}-{\left(\frac{{\dot{m}}^{h}}{\rho_{0}^{f}},q^{h}\right)}_{\Omega_{0}}+{\left(v^{\prime },H^{h}\nabla q^{h}\right)}_{\Omega_{0}},$$

(65d) $$0={\left(\frac{{\dot{m}}^{h}}{\rho_{0}^{\text{f}}},r^{h}\right)}_{\Omega_{0}}+{\left(K_{0}\nabla p^{h},\nabla q^{h}\right)}_{\Omega_{0}}+{\left(K_{0}^{m}\nabla m^{h},\nabla q^{h}\right)}_{\Omega_{0}}+{\langle W\cdot n,\;r^{h}\rangle }_{\Gamma }-{\left(S,q^{h}\right)}_{\Omega_{0}},$$

(65e) $$v^{\prime }=-\tau \left({\dot{v}}^{h}+\frac{1}{\rho_{0}^{\text{s}}}H^{h}\nabla p^{h}-b\right),$$

in which the terms corresponding to the deviatoric component of the stress tensor disappear because we have specialized the formulation to piecewise linear shape functions, and the vector ***W*** corresponds to the Lagrangian perfusion velocity. Similar to Eq. (60), we discretize Eq. (65) in time using the BDF2 scheme and solve the resulting system of equations for the velocity, pressure, and added mass using Newton’s method. We then update the displacement explicitly.

### 3.4. Choice of the stabilization parameter

For simplicity, the stabilization matrix ***τ*** is chosen to be isotropic [33], i.e., ***τ*** = *τ***I**. The stabilization parameter *τ* is an intrinsic time scale that can be obtained in various ways [31]. In our formulation, we choose the stabilization time scale between the time scale of the shear wave *Δt_μ_* of the elastic material and the time scale related to the time discretization *Δt*, such that

(66) $$\tau =\frac{c_{\tau }}{2}\max\left[\frac{\Delta t_{\mu }}{100},\min\left(\Delta t_{\mu },\;\Delta t\right)\right],\;c_{\tau }\in [0.01,0.03],\;\Delta t_{\mu }=\underset{e\in {𝒯}^{h}}{\min}\frac{h^{e}}{c_{\mu }^{e}}.$$

Here, *h^e^* is the characteristic length of element *e*, $c_{\mu }^{e}$ is the shear wave velocity, and ${𝒯}^{h}$ is the general triangulation. Although the stabilization term adds some numerical dissipation, its contribution is small, thanks to the choice of the stabilization parameter in the D-VMS method. This was demonstrated in [31,33] and shown in the numerical results in the following sections. Further, for the D-VMS method, numerical dissipation is necessary to satisfy the LBB criterion. The expression for *τ* (Eq. (66)) was obtained in previous studies [31,33] after intensive numerical experiments to minimize the numerical dissipation while maintaining stabilization. In the expression (Eq. (66)), the term min(*Δt_μ_*,*Δt*) is chosen so that the numerical dissipation is not too large to cause catastrophic failures in computation in the limit *Δt* → ∞. The term *Δt_μ_/*100 is introduced so that the numerical dissipation is stable in the limit *Δt* → 0. Further, to reduce excessive numerical dissipation for incompressible case, Rossi et al. [33] introduced the value of *c_τ_* in the range [0.01, 0.03] after numerical experimentation to maintain the stabilization, and we follow this approach here.

For isotropic materials, the shear wave velocity $c_{\mu }^{e}$ is [33]

(67) $$c_{\mu }^{e}=\sqrt{\left(W_{1}+W_{2}\right)/\rho_{0}^{\text{s}}},$$

in which $W_{1}=\frac{\partial W}{\partial {\bar{I}}_{1}}$ and $W_{2}=\frac{\partial W}{\partial {\bar{I}}_{2}}$. For anisotropic materials, we extend this definition of shear wave velocity using the anisotropic invariants. Here, we consider anisotropicity described in terms of two material directions (in the myocardium, fibre and sheet directions) and their interactions. Consequently, the shear wave velocity for the anisotropic material is

(68) $$c_{\mu }^{e}=\sqrt{\left(W_{1}+W_{2}+W_{4\text{f}}+W_{4\;\text{s}}+W_{8\;\text{s}}\right)/\rho_{0}^{\text{s}}},$$

in which $W_{4\text{f}}=\frac{\partial W}{\partial {\bar{I}}_{4\text{f}}}\;$, $W_{4s}=\frac{\partial W}{\partial {\bar{I}}_{4s}}\;$, and $W_{8\text{fs}}=\frac{\partial W}{\partial {\bar{I}}_{8\text{fs}}}\;$.

### 3.5. Choice of time step size

Even though the implicit time integration is unconditionally stable, for strongly nonlinear problems, the time step size choice is closely related to the convergence of nonlinear iterations. For the nonlinear convergence, an empirical relationship is obtained for the time step size as proportional to the velocity of the shear wave,

(69) $$\Delta t=\alpha_{\text{CFL}}\underset{e\in {𝒯}^{h}}{\min}\frac{h^{e}}{c_{\mu }^{e}},$$

in which *α*_CFL_ is the CFL number. In our numerical experiments, when we use 0.1 ≤ *α*_CFL_ ≤ 1, the nonlinear solver generally requires at most five Newton iterations. Note that stabilization method and the assumptions Eqs. (57) and (64) guarantee an accurate solution only if the mesh size and time step size are related by the CFL condition (Eq. (69)).

## 4. Results

Numerical tests are presented for benchmark test cases in two and three dimensions for both hyperelastic and poroelastic problems. In addition, the method’s capability is demonstrated by simulating LV dynamics and perfusion in an anatomical model derived from human image data.

### 4.1. Elastodynamics

This section details numerical tests of the anisotropic hyperelastic formulation, including a two-dimensional anisotropic compressed block, three-dimensional shear deformation, three-dimensional bending column, and three-dimensional twisting column.

#### 4.1.1. Hyperelastic anisotropic compressed block

**Fig. 1.**
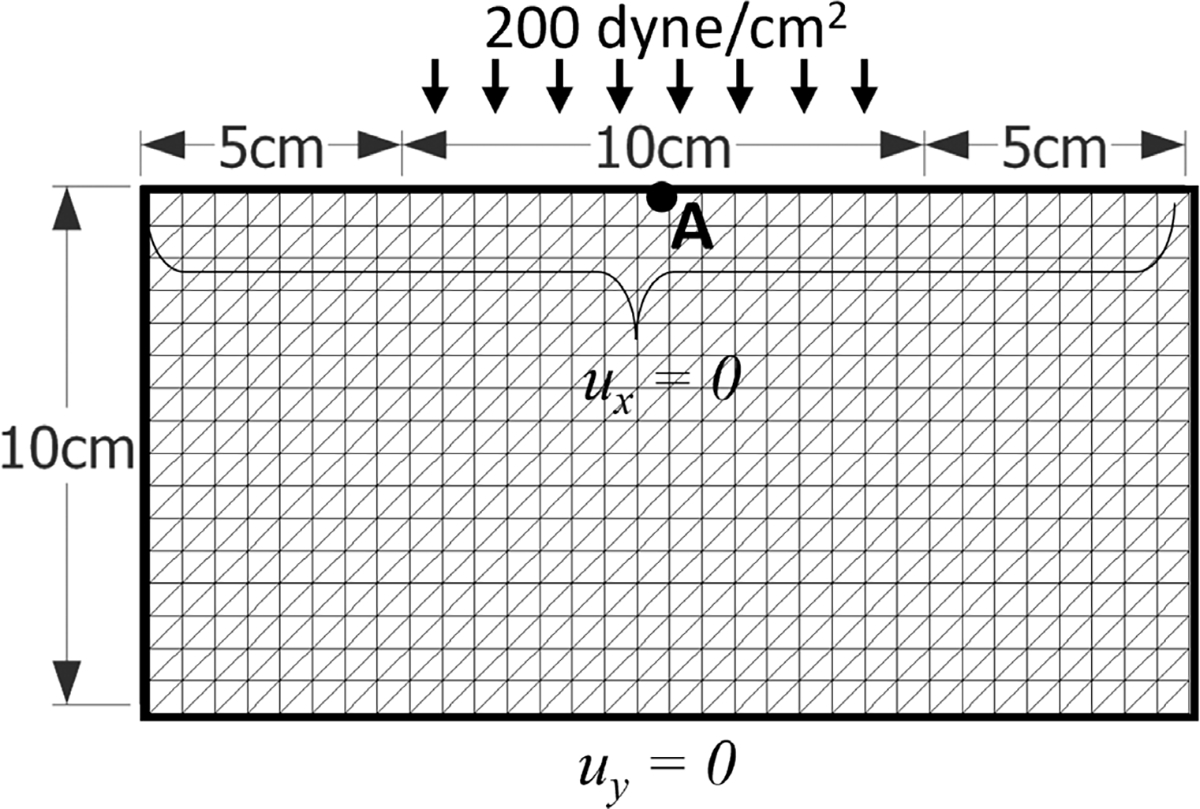
Computational setup for the compressed block test case (Section 4.1.1). The vertical displacement is constrained on the bottom surface and horizontal displacement is constrained on the top surface. A constant vertical loading of 200 dyne/cm^2^ is applied on the top mid-surface.

Fig. 1 shows the computational domain and boundary conditions for a two-dimensional compressed block benchmark with uniformly distributed vertical loading at the mid-portion of the top surface and traction-free boundary conditions on the left and right faces. The vertical displacement is constrained on the bottom surface, and the horizontal displacement is constrained on the top. We consider the standard reinforced model (Eq. (35)) with *G* = *G*_f_ = 80.194 dyne/cm^2^ and an initial fibre direction **f**_0_ = (0.866, 0.5). A constant density of $\rho_{0}^{\text{s}}=1.0\;\text{g}/{\text{cm}}^{3}$ is considered. Constant vertical loading of 200 dyne/cm^2^ is applied on the top mid-surface. Computations were carried out for eight sets of uniform meshes, with coarsest mesh spacing *h* = 5 cm (with *Δt* = 0.1s) and finest mesh spacing *h* = 0.2 cm (with *Δt* = 0.004s).

**Fig. 2.**
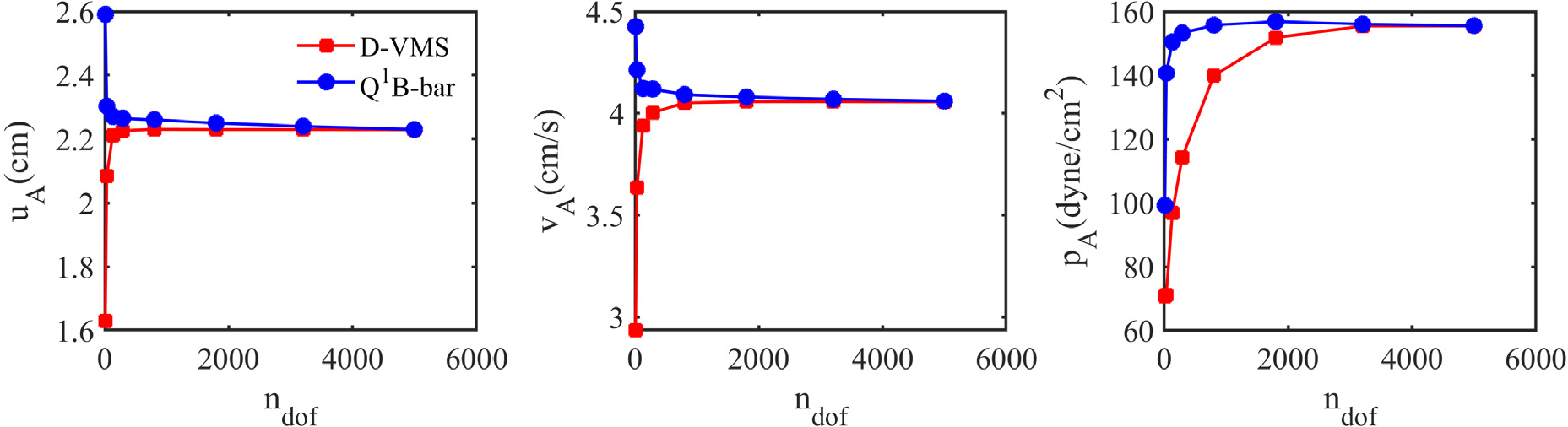
Convergence for the displacement *u*_A_, velocity *v*_A_, and pressure *p*_A_ at the midpoint on the top surface of the anisotropic compressed block obtained using the D-VMS method and *Q*^1^–*B*-bar method at *t* = 1.0s.

**Fig. 3.**
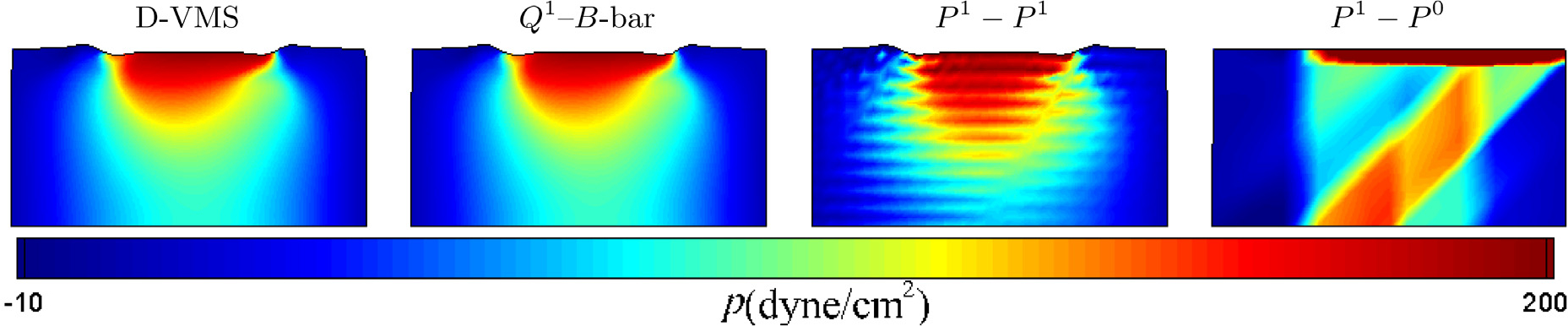
Pressure fields for the two-dimensional anisotropic compressed block obtained using the D-VMS method, *Q*^1^–*B*-bar method, *P*^1^ − *P*^1^ method, and *P*^1^ − *P*^0^ method at *t* = 0.1s for the finest mesh spacing *h* = 0.2 cm.

The results obtained from the D-VMS method are first compared with the standard *Q*^1^ finite element with reduced selective integration (*Q*^1^−*B*-bar) method [23], *P*^1^ − *P*^1^ (piecewise linear interpolation for displacement, velocity and pressure) method, and the *P*^1^ − *P*^0^ (piecewise linear interpolation for displacement and velocity and piecewise constant interpolation for pressure) method. Fig. 2 compares the grid convergence for the displacement, velocity, and pressure for the D-VMS and *Q*^1^−*B*-bar methods. It is clear from the figure that the displacement, velocity and pressure converge with grid refinement. Further, Fig. 3 shows that the pressure field obtained using the D-VMS method is smooth and in agreement with the *Q*^1^−*B*-bar method. Thus, it is evident that D-VMS produces a solution that is free of locking with a non-oscillatory pressure field. Note that the *P*^1^ − *P*^0^ locks, and the *P*^1^ − *P*^1^ formulation appears to avoid locking but generates an oscillatory pressure field.

**Fig. 4.**
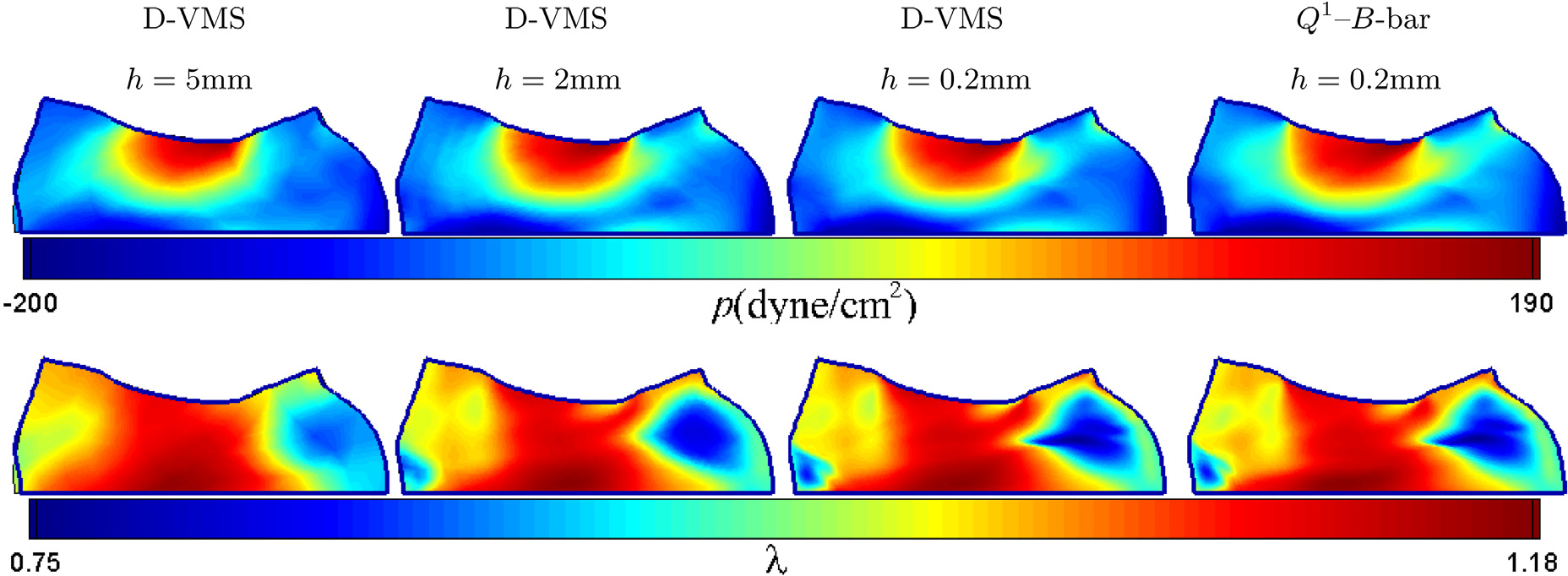
Pressure fields (top) and fibre stretch fields (bottom) for the anisotropic compressed block obtained using the D-VMS method with various mesh spacing and *Q*^1^–*B*-bar method at *t* = 1.0s. The thick blue line in each panel show the shape obtained using the *Q*^1^–*B*-bar method.

Fig. 4 shows the convergence for the pressure *p* and the fibre stretch $\lambda \;\left(\sqrt{I_{4\text{f}}}\right)$, for various mesh sizes and its comparison with the pressure field obtained by the *Q*^1^–*B*-bar method at *t* = 1.0s. The thick blue line in each figure represents the deformed shape obtained from the *Q*^1^–*B*-bar method. The fields of pressure and fibre stretch and the shapes of the deformed cube are in excellent agreement for the finest mesh spacing.

**Fig. 5.**
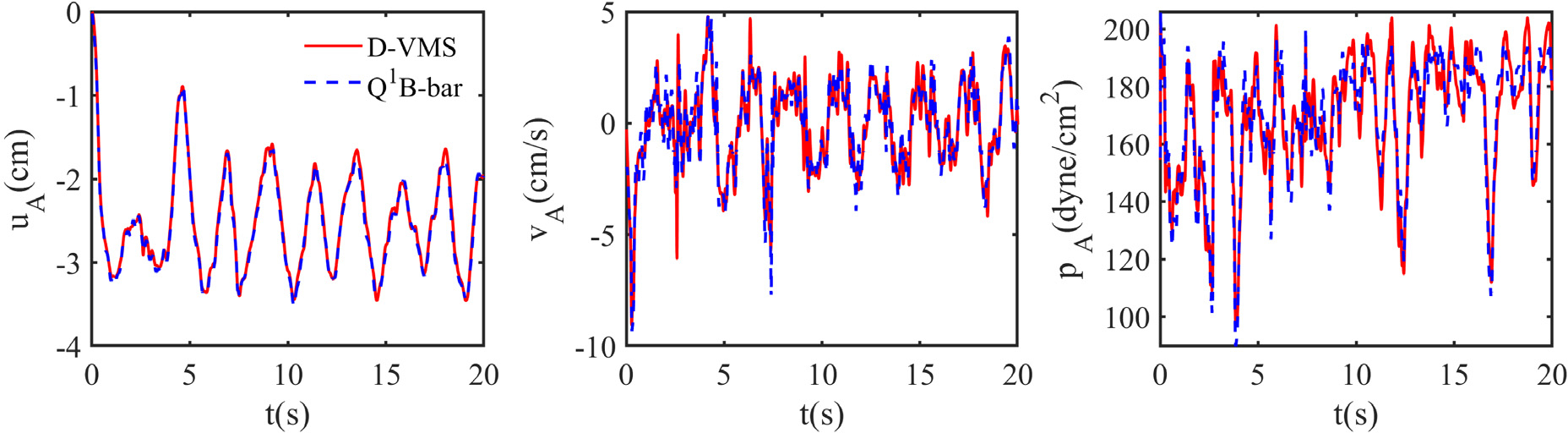
Temporal variation of the displacement *u*_A_, velocity *v*_A_, and pressure *p*_A_ at the midpoint on the top surface of the anisotropic compressed block obtained using the D-VMS method and *Q*^1^–*B*-bar method.

Fig. 5 shows the temporal variation of the displacement (*u*_A_), velocity (*v*_A_), and pressure (*p*_A_) at the point *A* indicated in Fig. 1 obtained from the D-VMS method and the *Q*^1^–*B*-bar method. The results are in excellent agreement for the displacement, velocity, and fibre stretch. The pressure is slightly more oscillatory for the stabilized method as compared to the *Q*^1^–*B*-bar method.

#### 4.1.2. Hyperelastic anisotropic shear

We now consider a hyperelastic material occupying the domain *Ω* = [0, 1 m] × [0, 1 m] × [0, 1 m] in which forcing is applied to obtain the exact shear deformation

(70) $$\begin{array}{l} u_{X}^{*}(X,t)=\alpha Z^{2}\sin(t),\\ u_{Y}^{*}(X,t)=\beta Z\sin(t),\\ u_{Z}^{*}(X,t)=0, \end{array}$$

where $u_{X}^{*}$, $u_{Y}^{*}$, and $u_{Z}^{*}$. are the displacements in *X*, *Y*, and *Z* directions, and *α* and *β* are constants.

For an anisotropic cube modelled by the standard-reinforced model (Eq. (35)) with fibre direction $f_{0}=\frac{1}{\sqrt{3}}(1,1,1)$, the resulting fibre stretch is

(71) $$\lambda^{*}(X,t)=\frac{\sqrt{(12\alpha Z\;+\;6\beta )\sin(t)\;+\;\left(-12\alpha^{2}Z^{2}\;-\;3\beta^{2}\right)\cos^{2}(t)\;+\;12\alpha^{2}Z^{2}\;+\;3\beta^{2}\;+\;9}}{3}.$$

The exact solution for the pressure is

(72) $$\begin{array}{l} \;p^{*}(X,t)=-\frac{10}{3}\left(\left(\frac{3\rho_{0}^{\text{f}}Z^{2}}{20}+G+\frac{2G_{\text{f}}}{3}\right)\alpha^{2}+\frac{3\beta^{2}\rho_{0}^{\text{f}}}{20}\right)Z^{2}\sin^{2}(t)+\\ \frac{1}{18}\left(\left(18XZ^{2}\rho_{0}^{\text{f}}-16ZG_{\text{f}}+36\left(G+\frac{2G_{\text{f}}}{3}\right)X\right)\alpha +18\rho_{0}^{\text{f}}\beta ZY\right)\sin(t). \end{array}$$

Appendix A provides the detailed derivation of the exact solution. For the computation, we use initial and boundary conditions obtained from the imposed exact solution. For the bottom plane *Z* = 0, the zero velocity boundary condition is used. For all other faces, traction conditions obtained from the exact solution of the PK2 stress are used. The remaining physical parameters used are $\rho_{0}^{\text{s}}=1.0\;\;\text{kg}/{\text{m}}^{3}$, *G* = 1.0 Pa, *G _f_* = 1.0 Pa, *α* = 0.1, and *β* = 0.1.

**Fig. 6.**
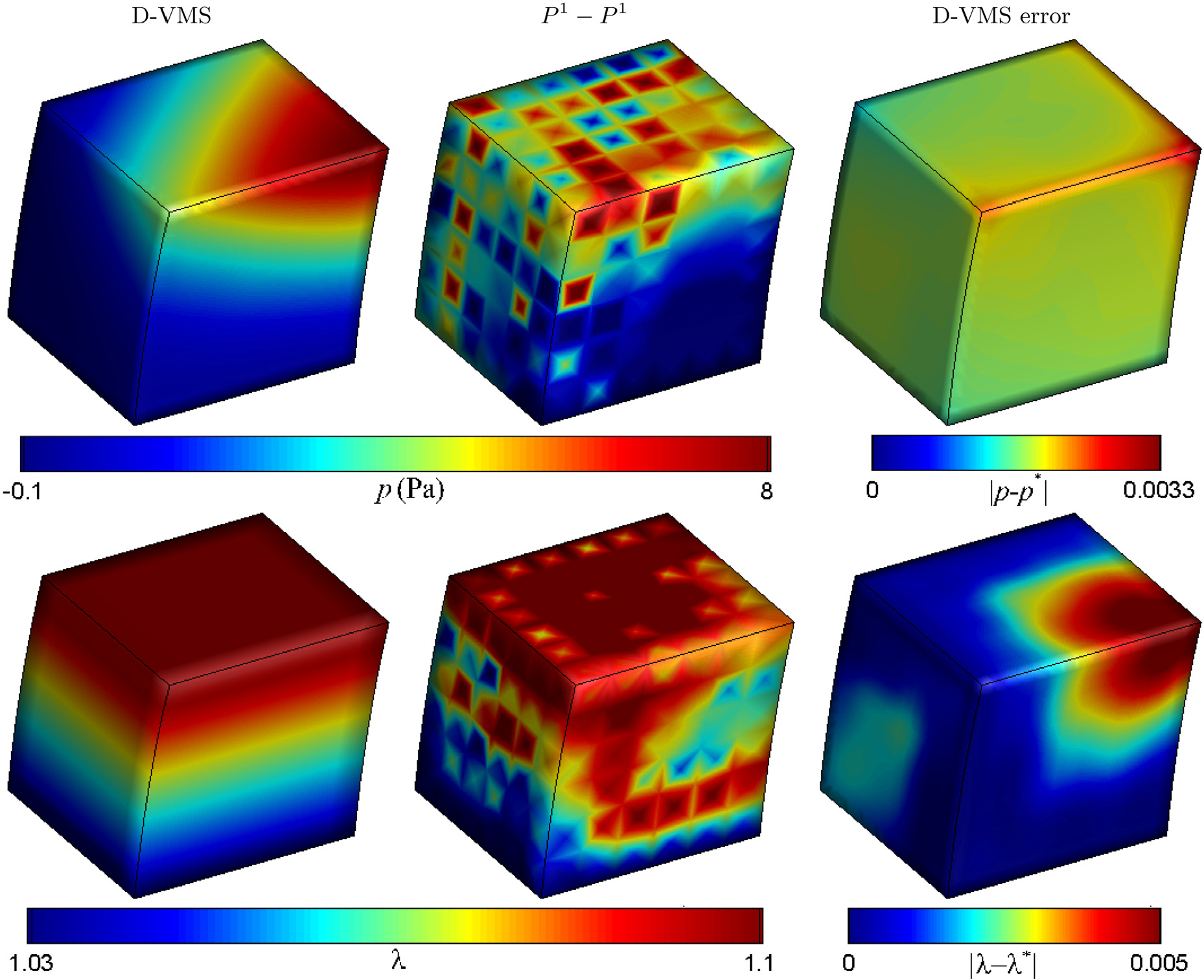
Pressure fields (top) and fibre stretch fields (bottom) obtained using the D-VMS method and *P*^1^ − *P*^1^ method along with the absolute errors in the pressure and fibre stretch at *t* = 0.25s for the shear deformation of the anisotropic cube (Section 4.1.2).

Fig. 6 shows the pressure obtained from the numerical simulation using the D-VMS and *P*^1^ − *P*^1^ methods and the error in the pressure at *t* = 0.25s. The simulations are carried out for meshes with mesh spacing *h* = 0.167m and time step size *Δt* = 0.05s. The pressure field obtained using the D-VMS method is less oscillatory than the *P*^1^ − *P*^1^ method. Fig. 6 also shows the fibre stretches obtained using the D-VMS and *P*^1^ − *P*^1^ methods and the error in fibre stretch obtained using the D-VMS method. The maximum value of the error in pressure and the fibre stretch are found to be 0.0033Pa and 0.005, respectively. Similar to the pressure, the fibre stretch is also oscillatory for the *P*^1^ − *P*^1^ method compared to the D-VMS method.

**Fig. 7.**
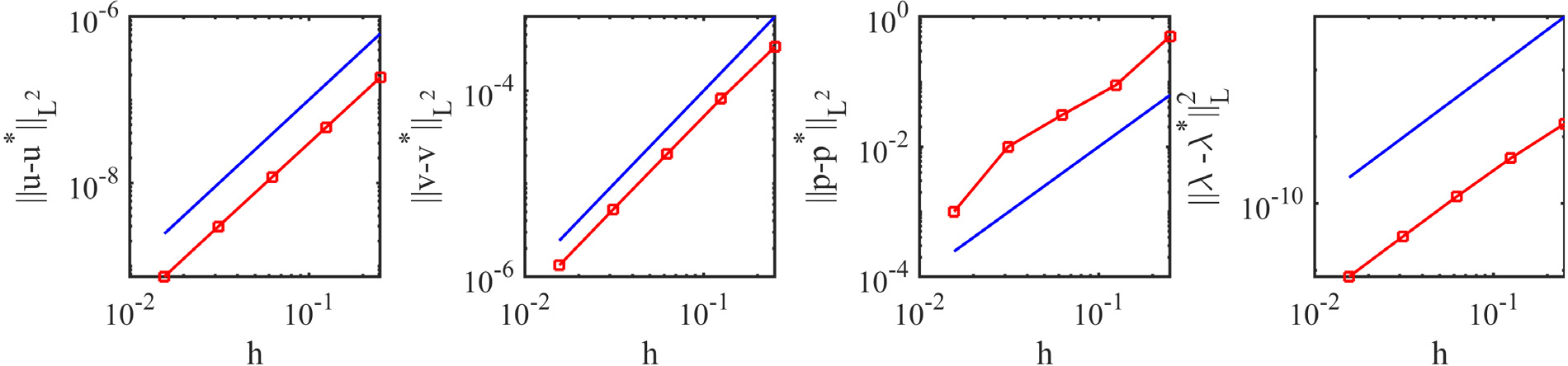
*L*^2^ errors for the displacement, velocity, pressure, and fibre stretch for the shear deformation of the anisotropic cube at *t* = 10.0s. The blue line in each figure indicates second-order convergence (∝ *h*^2^).

A convergence study was performed using a set of five grids with the coarsest mesh spacing of *h* = 0.25m (with time step size *Δt* = 0.1s) and the finest mesh spacing of *h* = 0.015625m (with time step size *Δt* = 0.00625s). Fig. 7 shows the convergence of displacement, velocity, pressure, and fibre stretch. Similar to an earlier isotropic convergence study [33], a second-order convergence is observed for all variables. This illustrates that the D-VMS method can be extended to anisotropic cases without loss in accuracy.

#### 4.1.3. Hyperelastic anisotropic bending column

**Fig. 8.**
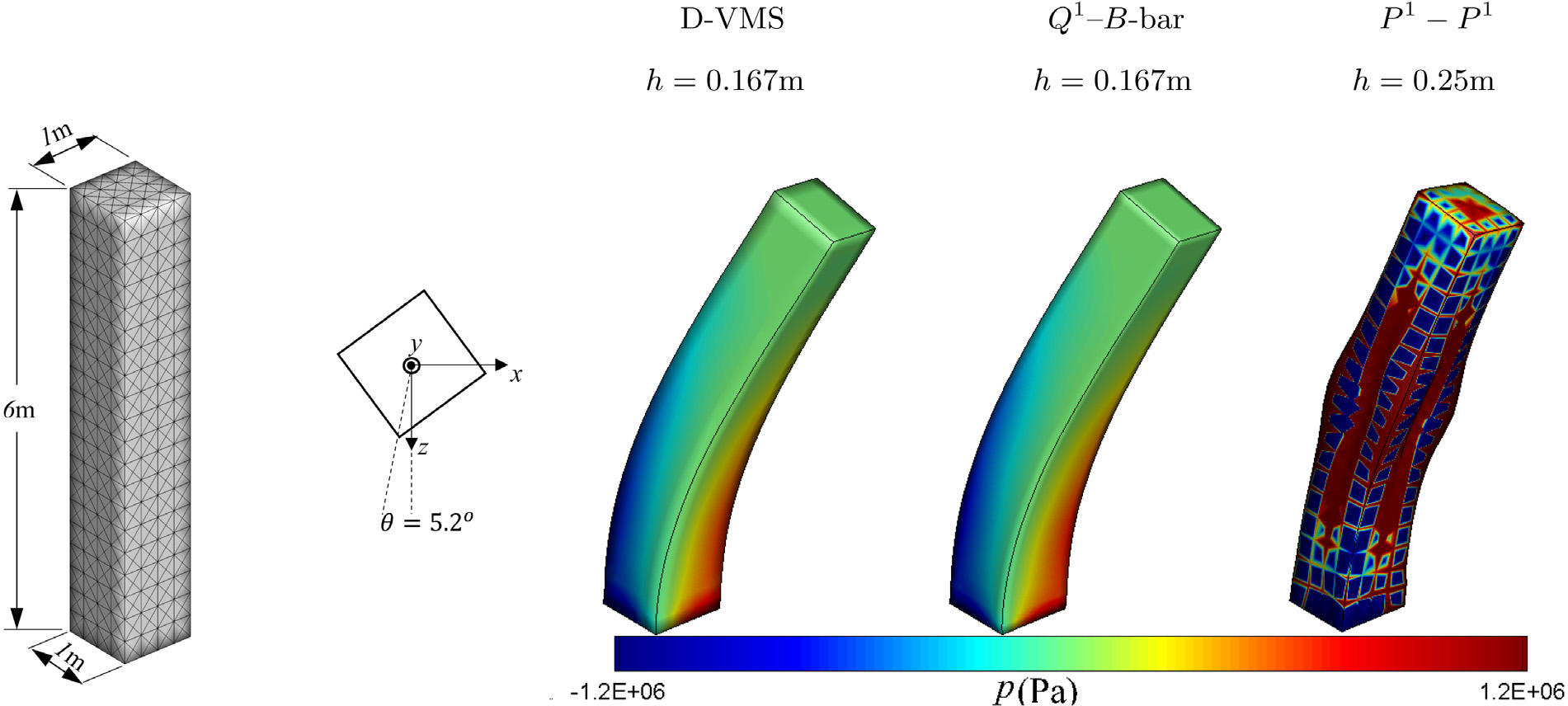
Left: Computational domain for the bending column (Section 4.1.3). The bottom plane *Z* = 0 is fixed and the axis of bending is asymmetric so that the solution is not axisymmetric. Right: Pressure fields for the three-dimensional anisotropic bending column problem obtained using the D-VMS method, *Q*^1^–*B*-bar method, and *P*^1^ − *P*^1^ method at *t* = 0.2s.

We next consider a three-dimensional anisotropic bending column. Fig. 8 shows the computational domain for the bending column. The bending axis is slightly inclined such that the solution is not symmetric. A fixed displacement boundary condition is used for the bottom plane *Y* = 0, and a traction-free boundary condition is used for all the other faces. The initial velocity is set to

(73) $$v(X,\;0)=\left(\frac{5}{3}Y,\;0,\;0\right)\text{m}/\text{s}.$$

We use the standard reinforced model (Eq. (35)) with *G* = *G*_f_ = 5.67 × 10^6^Pa, initial fibre direction **f**_0_ = (0, 0.5, 0.866), and $\rho_{0}^{s}=1100\;\text{kg}/{\text{m}}^{3}$. Simulations are performed using the D-VMS method, *P*^1^ − *P*^1^ method, and *Q*^1^–*B*-bar method until *t* = 0.5s. For the D-VMS method, the simulations are carried out for three different tetrahedral meshes with mesh spacing *h* = 0.5m (with time step size *Δt* = 0.005s), *h* = 0.25m (with time step size *Δt* = 0.0025s) and *h* = 0.167m (with time step size *Δt* = 0.001675s), respectively. For the *P*^1^ − *P*^1^ method, the simulations are carried out for the tetrahedral mesh with mesh spacing *h* = 0.25m (with time step size *Δt* = 0.0025s). For the *Q*^1^–*B*-bar method, hexahedral mesh with mesh spacing *h* = 0.167m (with time step size *Δt* = 0.001675s) is used.

Fig. 8 shows the pressure fields in the deformed configuration obtained from the D-VMS method, *Q*^1^–*B*-bar method, and *P*^1^ − *P*^1^ method. It is clear from the figure that the *P*^1^ − *P*^1^ method generates highly oscillatory pressures. The D-VMS method results in smooth pressure distribution, and the results are in agreement with the *Q*^1^–*B*-bar method.

**Fig. 9.**
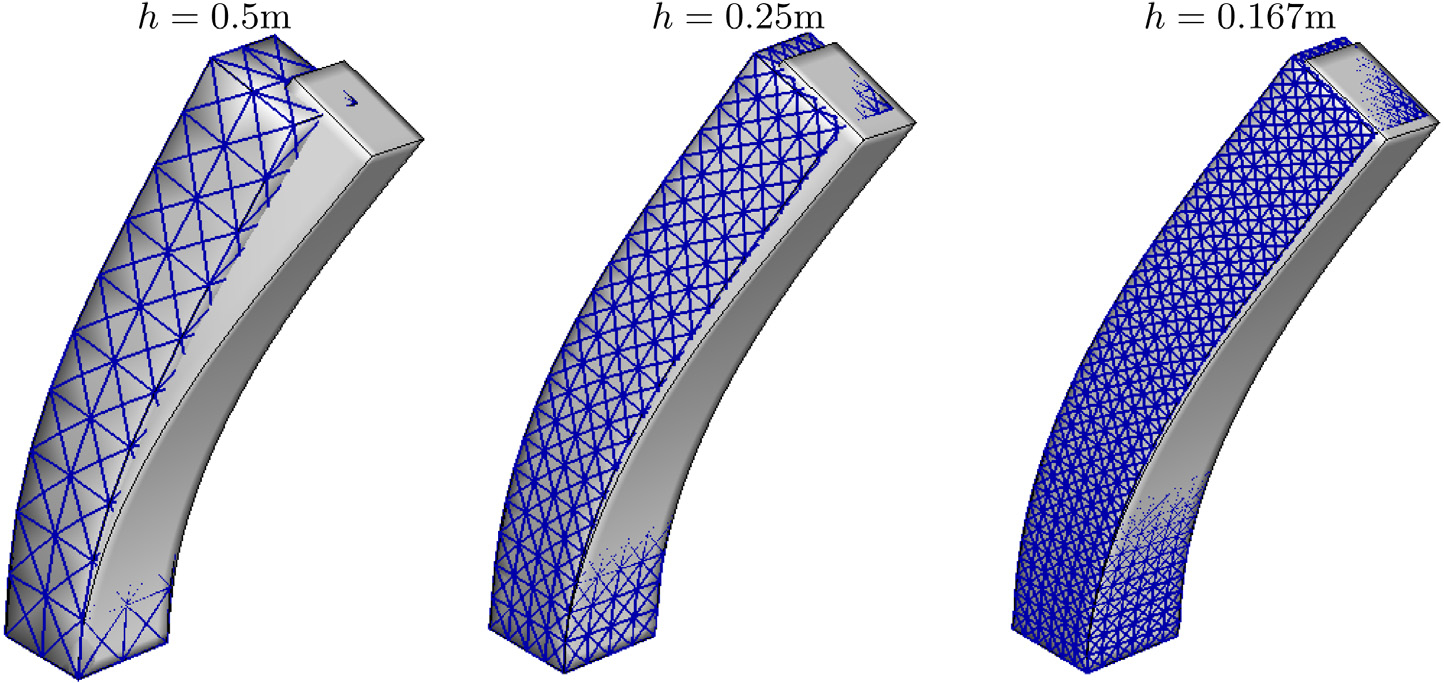
Deformed shapes of the three-dimensional anisotropic bending column obtained using the D-VMS method (shown with mesh) with various mesh spacing superimposed with the shape obtained using the *Q*^1^–*B*-bar method (with *h* = 0.167m) at *t* = 0.5s.

Fig. 9 shows the deformed shape of the bending column for various mesh sizes obtained from the D-VMS method and its comparison with the results obtained from the *Q*^1^–*B*-bar method with a finer mesh size. The figure shows that the results obtained using the finer meshes match the D-VMS and *Q*^1^–*B*-bar methods.

#### 4.1.4. Hyperelastic anisotropic twisting column

Next, we consider a twisting column, with the computational domain, boundary conditions, and the fibre direction the same as the bending column, shown in Fig. 8, but with initial velocity

(74) $$v(X,0)=100\;\sin\left(\frac{\pi Y}{12}\right)(\begin{array}{lll} Z, & 0, & -X)\text{m}/\text{s} \end{array}.$$

**Fig. 10.**
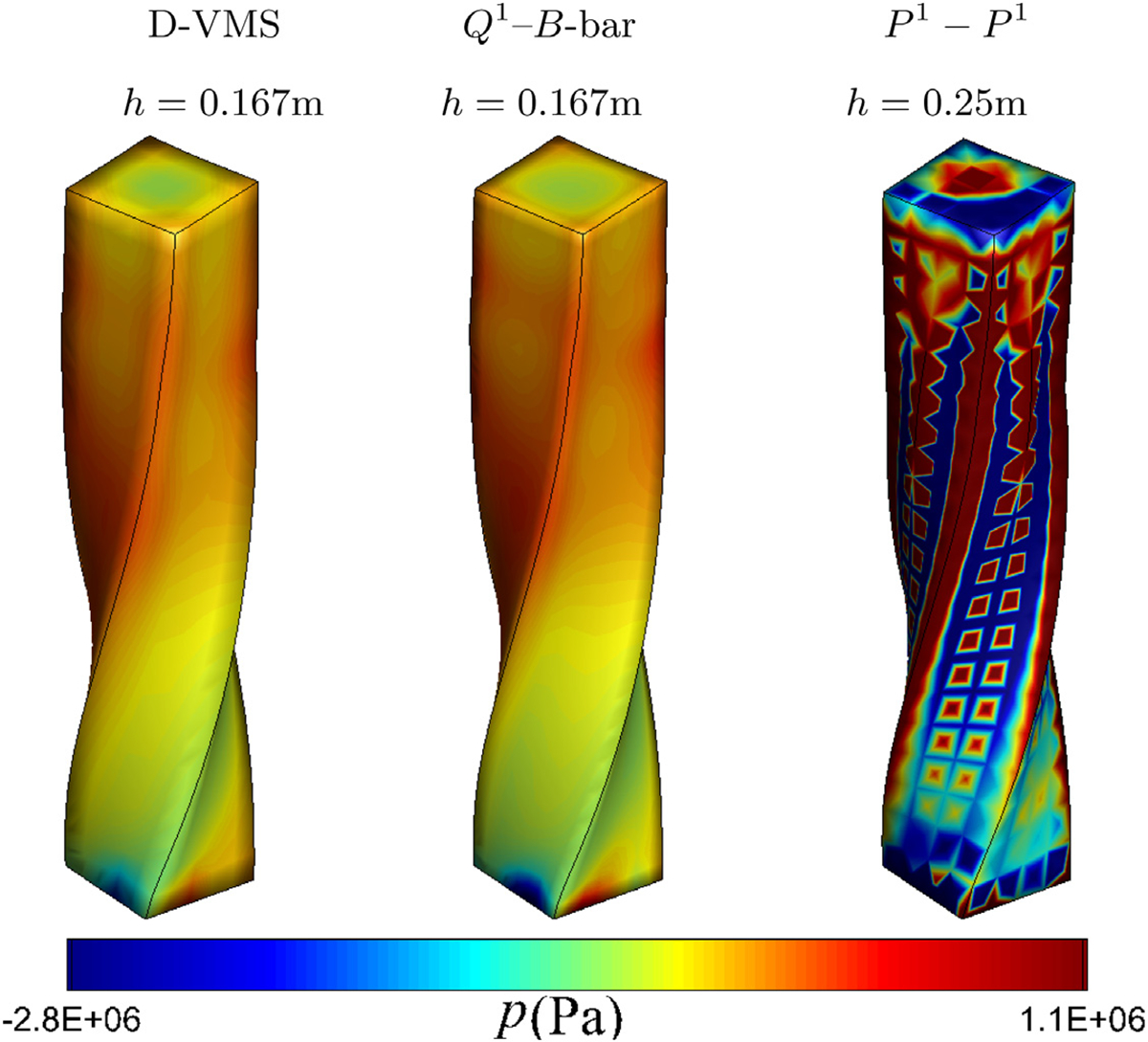
Pressure fields for the three-dimensional anisotropic twisting column obtained using the D-VMS method, *Q*^1^–*B*-bar method, and *P*^1^ − *P*^1^ method at *t* = 0.02s (Section 4.1.4).

**Fig. 11.**
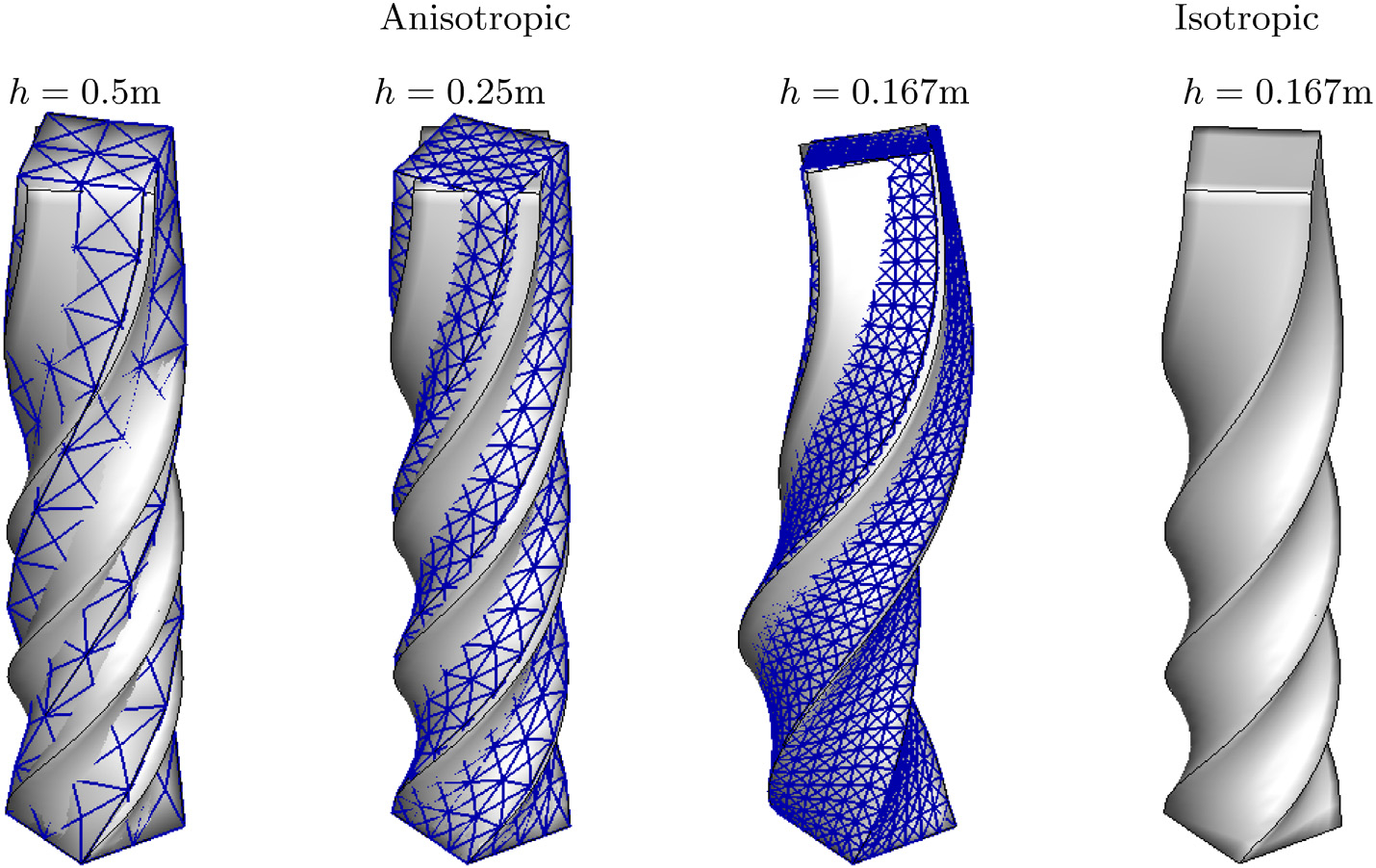
Deformed shapes of the three-dimensional anisotropic twisting column problem obtained using the D-VMS method (shown with mesh) with various mesh spacing superimposed with the shape obtained using the *Q*^1^–*B*-bar (with *h* = 0.167m) method and deformed shape of three-dimensional twisting column without fibres (*G*_f_ = 0) obtained using the *Q*^1^–*B*-bar method at *t* = 0.1s.

**Fig. 12.**
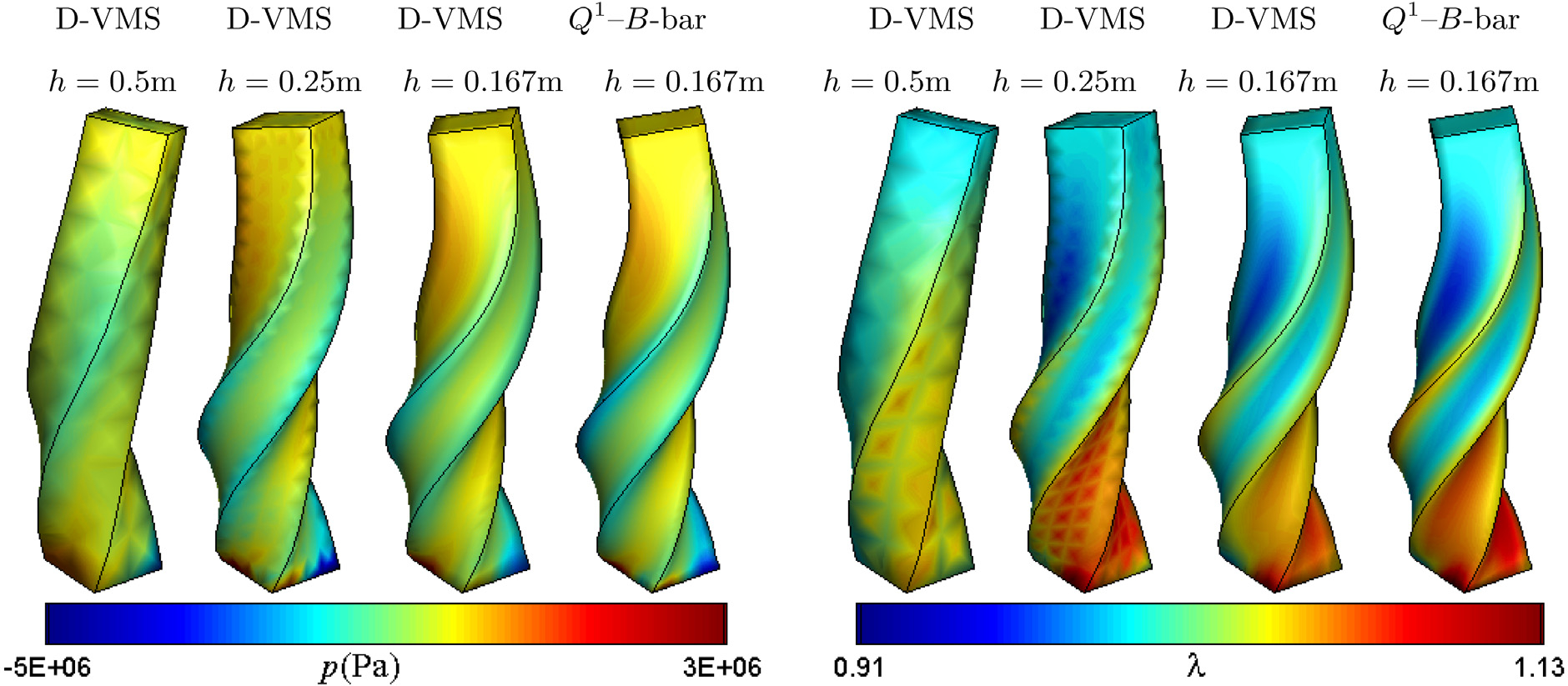
Pressure fields (left) and fibre stretch fields (right) for the three-dimensional anisotropic twisting column obtained using the D-VMS method with various meshes and the *Q*^1^–*B*-bar method at *t* = 0.1s.

All other physical parameters are the same as that of the bending column. The mesh for the grid convergence is also the same as the bending problem. Fig. 10 compares the pressure fields obtained from the various methods. Similar to the bending column, the pressures obtained from the *P*^1^ − *P*^1^ method are highly oscillatory. Fig. 11 shows that the result for the finer mesh obtained using the D-VMS method is in good agreement with that obtained from the *Q*^1^–*B*-bar method. Fig. 11 also shows the deformed shape of the twisting column without the fibres (*G*_f_ = 0). It is clear from the figure that the anisotropicity introduced asymmetric twisting due to the fibre reinforcement. Fig. 12 shows clear convergence of the pressure and the fibre stretch with refined grids.

### 4.2. Poroelastodynamics

This section presents numerical tests of the poroelastic formulation, including an anisotropic two-dimensional compressing block, isotropic three-dimensional shear deformation, and isotropic three-dimensional swelling and shrinking cubes.

#### 4.2.1. Anisotropic porous compressed block

We now consider a porous version of the anisotropic compressed block discussed previously with an initial porosity *ϕ*_0_ = 0.1 and a permeability **K** = 1.0 × 10^−5^**I** cm^4^dyne^−1^s^−1^. The source term is proportional to the pore pressure (*S* = *β*_so_ *p*^pore^). A source constant of *β*_so_ = 1.0 × 10^−5^ cm^2^dyne^−1^s^−1^ is used.

**Fig. 13.**
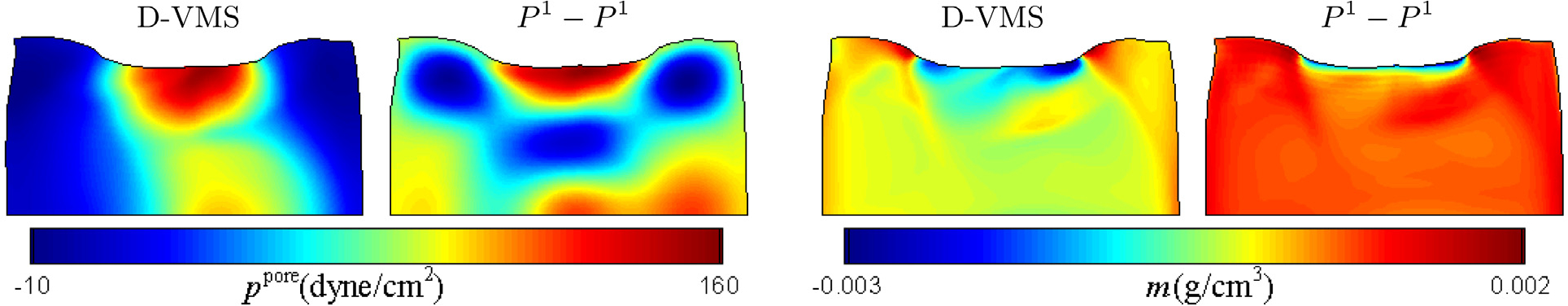
Pore pressure fields (left) and added mass fields (right) for the anisotropic porous compressed block obtained using the D-VMS method and *P*^1^ − *P*^1^ method at *t* = 0.3s with mesh spacing *h* = 0.2cm (Section 4.2.1).

**Fig. 14.**
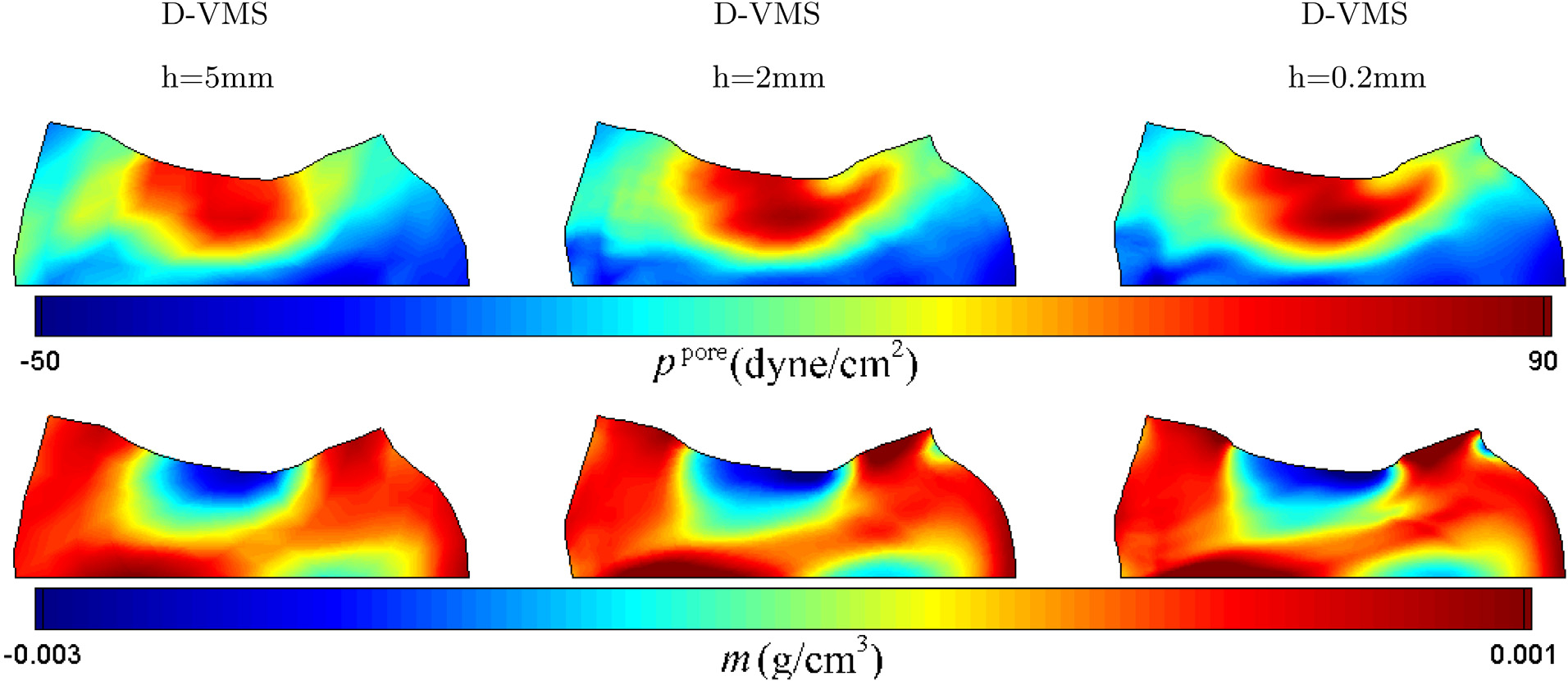
Pore pressure fields (top) and added mass fields (bottom) for the anisotropic porous compressed block obtained using different meshes at *t* = 1.0s.

**Fig. 15.**
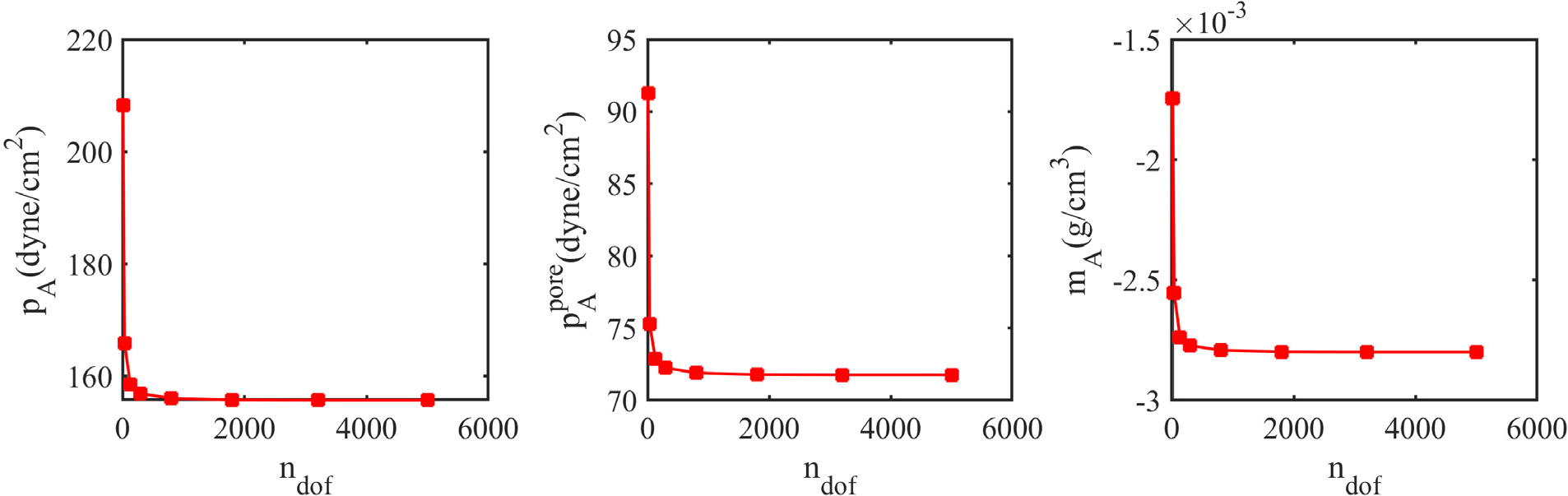
Convergence of the pressure *p*_A_, pore pressure $p_{\text{A}}^{\text{pore}}$, and added mass *m*_A_ at the midpoint on the top surface for the anisotropic porous compressed block for various numbers of degrees of freedom at *t* = 1.0s.

Fig. 13 compares the fields of pore pressure and added mass obtained from the D-VMS method and the *P*^1^ − *P*^1^ method. The *P*^1^ − *P*^1^ results are clearly oscillatory, although they are less oscillatory as compared to the hyperelastodynamics case. This may be because the skeleton is slightly compressible for the poroelastic case due to the presence of the fluid as compared to the full incompressibility in the hyperelastic case. Fig. 14 shows that the pore pressure field *p*^pore^ and the fields of added mass *m* converge under mesh refinement. Fig. 15 shows the convergence of the pressure *p*, the pore pressure *p*^pore^, and the added mass *m* at the midpoint on the top surface of the compressed block.

#### 4.2.2. Isotropic porous shear

Next, we consider a three-dimensional shear deformation with poroelasticity. For elasticity, the isotropic neo-Hookean model (Eq. (33)) is used. We impose the shear motion

(75) $$\begin{array}{l} u_{X}^{*}(X,t)=\alpha XZ\sin(t),\\ u_{Y}^{*}(X,t)=\beta Z\sin(t),\\ u_{Z}^{*}(X,t)=0. \end{array}$$

To satisfy the mass balance condition of the skeleton, $J-1-\frac{m}{\rho_{0}^{f}}=0$, the exact solution for the added mass is

(76) $$m^{*}(X,t)=\rho_{0}^{\text{f}}\alpha Z\sin(t).$$

Using *p^c^* = 0, the pore pressure (Eq. (23)) is

(77) $$\begin{array}{l} p^{\text{pore*}\;}(X,t)=-\frac{2X^{2}\left(-\frac{3\rho_{0}^{\text{f}}Z{\left(1+\alpha Z\sin(t)\right)}^{\frac{5}{3}}}{4}+G\alpha \sin\;(t)\right)\sin(t)\;\alpha }{3{\left(1\;+\;\alpha Z\sin\;(t)\right)}^{\frac{5}{3}}}+\\ \frac{\left(\rho_{0}^{\text{f}}Z{\left(1\;+\;\alpha Z\sin\;(t)\right)}^{\frac{5}{3}}-\frac{2G\alpha \sin(t)}{3}\right)\sin\;(t)\beta Y}{{\left(1\;+\;\alpha Z\sin\;(t)\right)}^{\frac{8}{3}}}+\kappa \rho_{0}^{\text{f}}\alpha Z\sin\;(t). \end{array}$$

The detailed derivation of this expression is given in Appendix B.

For the numerical simulations, we use the initial condition ***v*** (***X***, 0) = v* (***X***, 0) and *m* (***X***, 0) = 0. The boundary conditions are the same as that of the hyperelastic shear deformation (Section 4.1.2). For the pore-fluid, *c* = 0 is used in the compaction penalization (Eq. (36)).

**Fig. 16.**
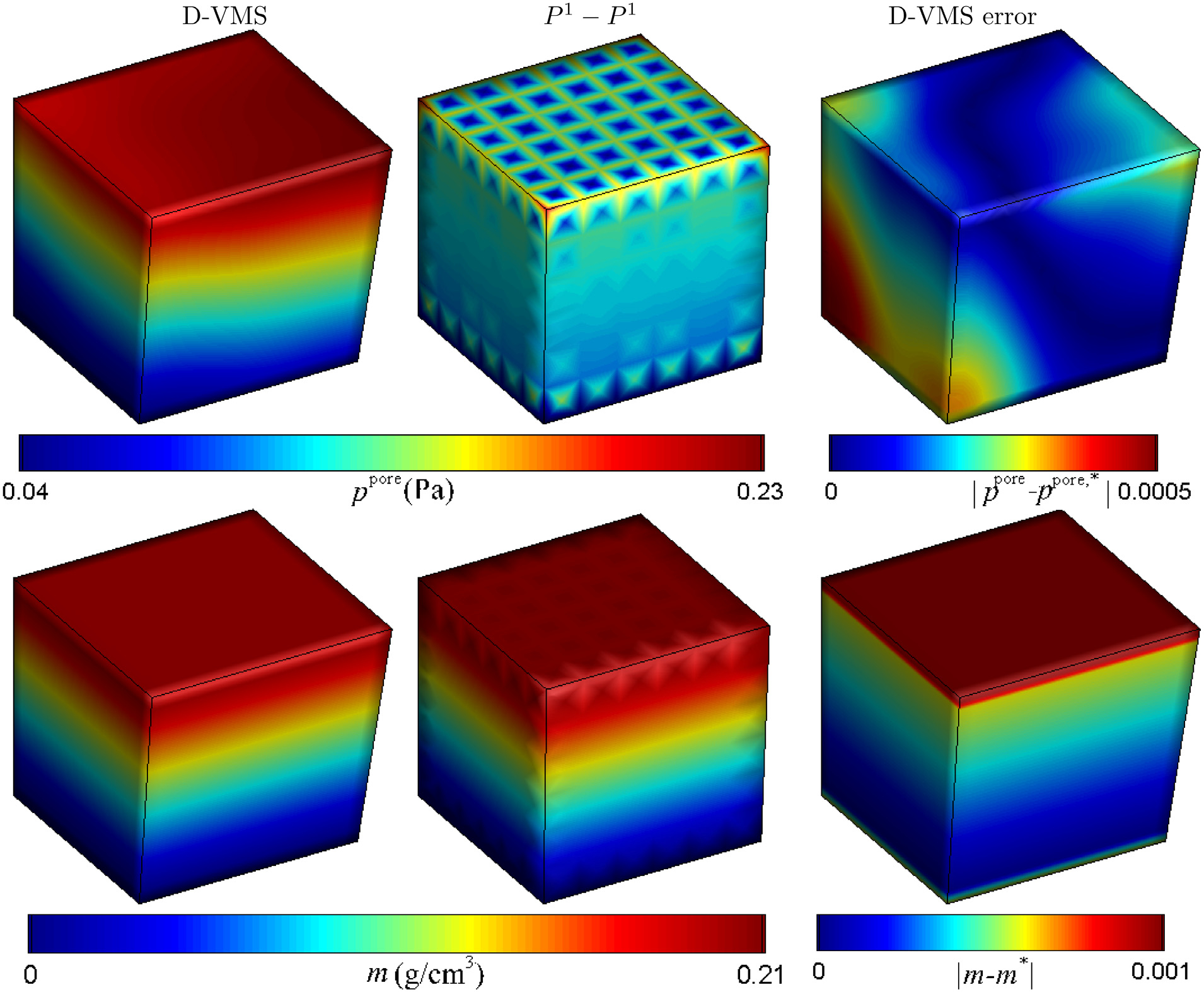
Pore pressure fields (top) and added mass fields (bottom) obtained using the D-VMS method and *P*^1^ − *P*^1^ method and absolute errors in the pore pressure and added mass at *t* = 0.25s for the shear deformation of the poroelastic isotropic cube (Section 4.2.2).

**Fig. 17.**
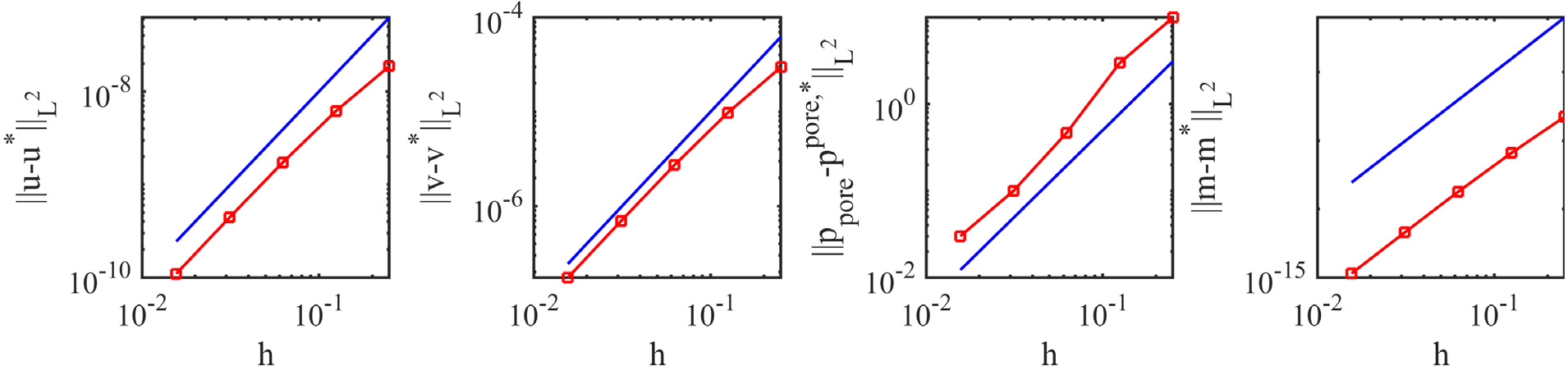
*L*^2^ errors for the displacement, velocity, pressure, and added mass for shear deformation of the three-dimensional isotropic poroelastic cube at *t* = 10.0s. The blue line in each figure indicates second-order convergence (∝ *h*^2^).

Fig. 16 compares the pore pressure fields and the added mass obtained from the *P*^1^ − *P*^1^ method and the D-VMS method. Similar to the hyperelastic case, the D-VMS method results in a smooth pressure field compared to the oscillatory pressure field obtained using the *P*^1^ − *P*^1^ method. Fig. 16 also shows a negligible difference between the results obtained from the simulation and the exact solution. Fig. 17 shows that the displacement, velocity, pore pressure, and added mass obtained using the D-VMS method are second-order accurate.

#### 4.2.3. Swelling and shrinking

**Fig. 18.**
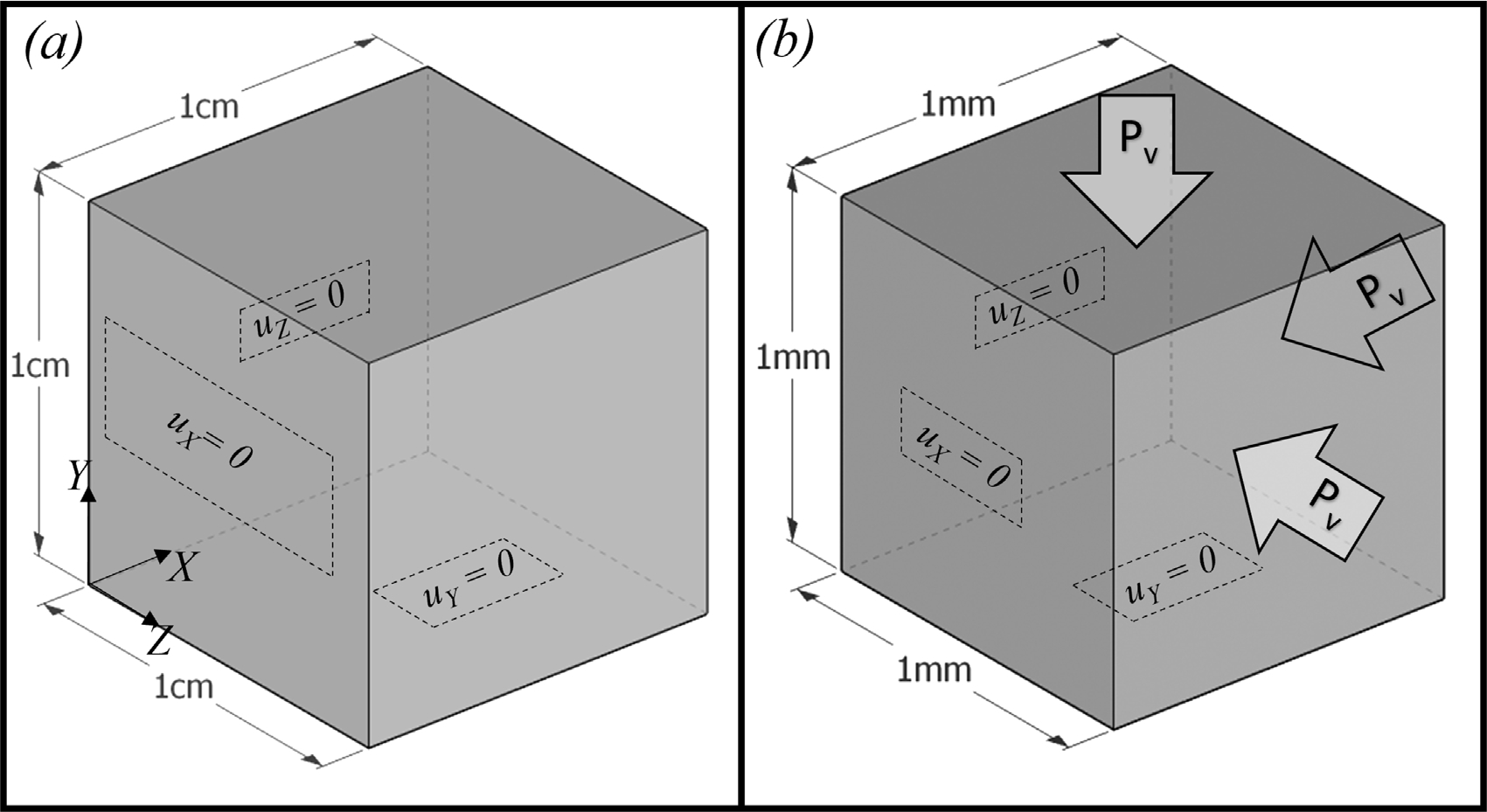
Computational setup along with boundary conditions for the verification of poroelastic problems of (a) a swelling poroelastic cube resulting from an applied pore pressure gradient, and (b) a shrinking poroelastic cube resulting from applied compressive forces (Section 4.2.3).

Here we show the performance of the D-VMS method on the two poroelastic test cases proposed by Chapelle et al. [11]. Fig. 18 shows the computational domains for the two cases: a swelling cube and a shrinking cube. For both cases, a Mooney–Rivlin model (Eq. (34)) is used for the hyperelastic part of the strain energy function, with solid and fluid densities $\rho_{0}^{\text{s}}=\rho_{0}^{\text{f}}=1000\;\text{kg}/{\text{m}}^{3}$, material constants *C*_1_ = 2 kPa and *C*_2_ = 0.033 kPa, initial porosity *ϕ*_0_ = 0.1, and pore pressure constant *κ*_s_ = 2 kPa. For the swelling and shrinking cases, the permeabilities of the fluid are taken as **K** = 10^−4^**I** m^2^kPa^−1^s^−1^ and **K** = 2.5 × 10^−3^**I** m^2^kPa^−1^s^−1^, respectively. The simulations are performed using a mesh with mesh spacing *h* = 0.05cm (and time step size *Δt* = 0.001s) for the swelling cube and *h* = 0.05 mm (and time step size *Δt* = 0.0001s) for the shrinking cube. For the first test case, the swelling of the cube is due to the pore pressure difference across two opposite faces of the cube (Fig. 18a). The pore pressure *p*^pore^ is gradually increased on the left surface (*X* = 0), and *p*^pore^ = 0 is applied to the right surface. The other four faces are set to be impermeable to constrain the flow to be in one direction, and no source/sink is used. Note that no external forces are acting on the cube for this problem. The deformation is entirely driven by the Darcy flow in the pore fluid. Chapelle et al. [11] used a pressure-based equation for Darcy flow, whereas we used the added mass-based formulation. Thus, for the cube swelling case, the pore pressure boundary conditions used by Chapelle et al. [11] are converted to boundary conditions for *m*. The added mass *m* on the left surface (*X* = 0cm) is set to increase gradually $\left(m(0,Y,Z,t)=0.5\left(1-\exp\left(-t^{2}/0.25\right)\right)\rho_{0}^{\text{f}}\right)$, and *m* on the right surface (*X* = 1.0cm) is kept fixed at zero (Fig. 18a).

For the second case, the shrinking of the cube is driven by external forces applied on all six faces of the cube. In this case, normal displacement is constrained on three faces (*X* = 0,*Y* = 0, *Z* = 0), and normal forces are applied on the other three faces (*X* = 1.0 mm,*Y* = 1.0 mm, *Z* = 1.0 mm). In addition, a pressure-dependent sink term, *S* = −*β*^si^(*p*^pore^ − *p*^si^), is imposed, and a zero normal gradient of *m* is applied on all the faces of the cube. For the numerical simulation, *β*^si^ = 0.01 kPa^−1^s^−1^ and *p*^si^ = 0 kPa are used. Note that for this case, the fluid added mass and the porosity are coupled with the normal solid pressure and the solid pressure and its deformation depends on the porosity (Eq. (30)). Thus, the problem serves to verify the two-way coupling between the solid deformation and the Darcy flow.

**Fig. 19.**
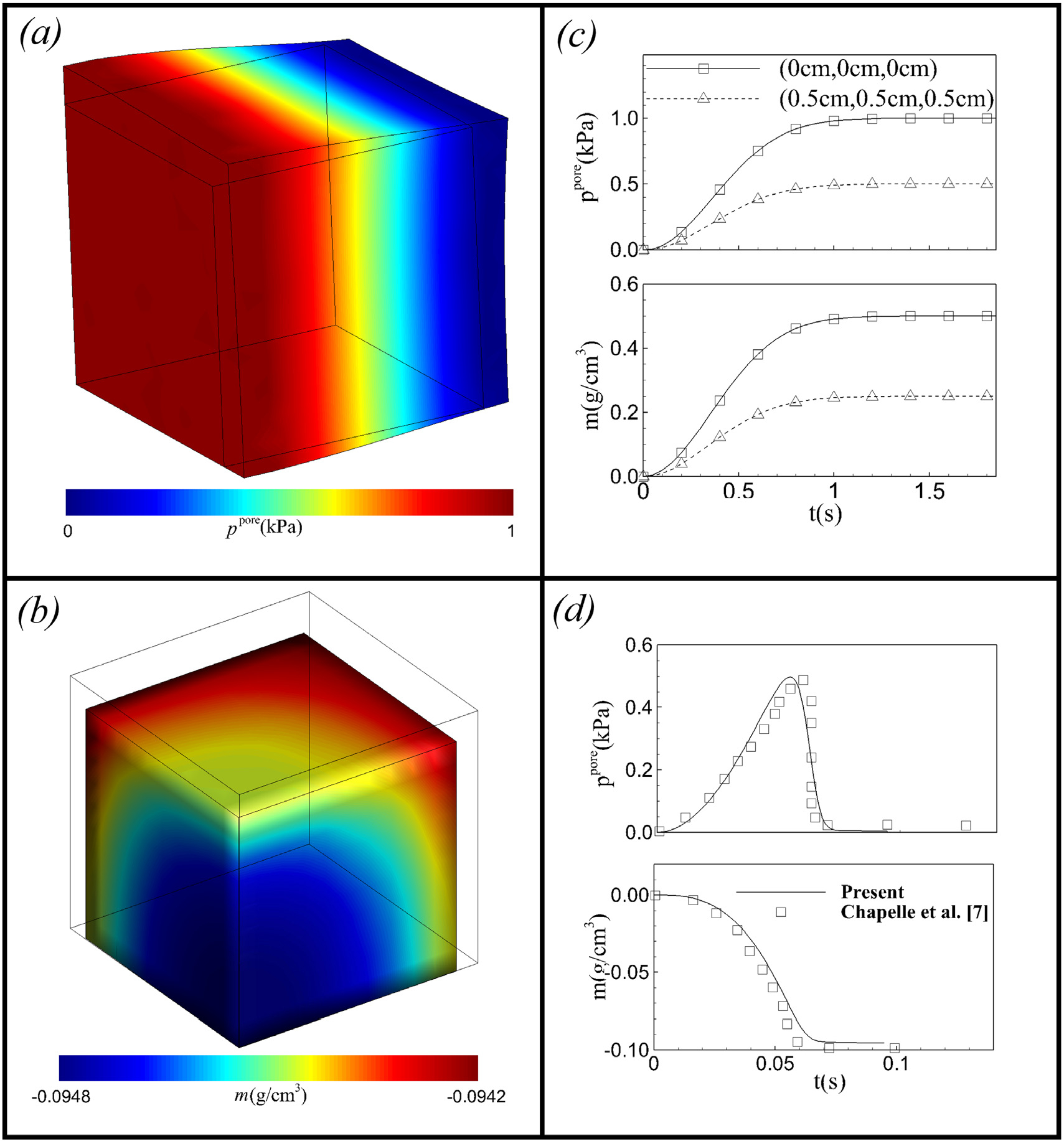
(a) Pore pressure field for the swelling cube and (b) the added mass field for the shrinking cube at steady state condition along with the temporal variation of *p*^pore^ and *m* at (c) the points (0 cm,0 cm,0 cm) and (0.5 cm,0.5 cm,0.5 cm) for the swelling cube and (d) for the point (0.5 mm,0.5 mm,0.5 mm) for the shrinking cube.

Fig. 19 shows the deformed position and fields for the total pore pressure *p*^pore^ (for the swelling cube) and added mass *m* (for the shrinking cube). For the swelling case, Fig. 19c shows that the gradient in *m* and pore pressure *p*^pore^ resulted in swelling of the cube near the left surface (*X* = 0) and fluid flow from the left to the right surface. The swelling gradually decreases from the left to the right surface. Fig. 19c shows an excellent agreement between our results and the literature for the transient variation of pore pressure *p*^pore^ and *m* at the middle point (0 cm,0 cm,0 cm) and the right corner point (0.5 cm,0.5 cm,0.5 cm).

For the shrinking case, Fig. 19d shows that the fluid in the cube is completely drained at a steady-state, resulting in a reduction in the volume of the cube by almost 10%. Fig. 19d also shows the transient variation of the pore pressure *p*^pore^ and *m* at the mid-point. The pressure increases gradually as per the applied pressure on the three surfaces. When the fluid is almost completely drained out (*m* ≈ −*ϕ*_0_), the penalty pressure *p*^c^ takes a very large negative value, and the total pore pressure *p*^pore^ suddenly drops to zero, restricting further drainage of fluid (Fig. 19d). The results are in very good agreement with the results from Chapelle et al. [11].

### 4.3. Perfusion in left ventricle

**Fig. 20.**
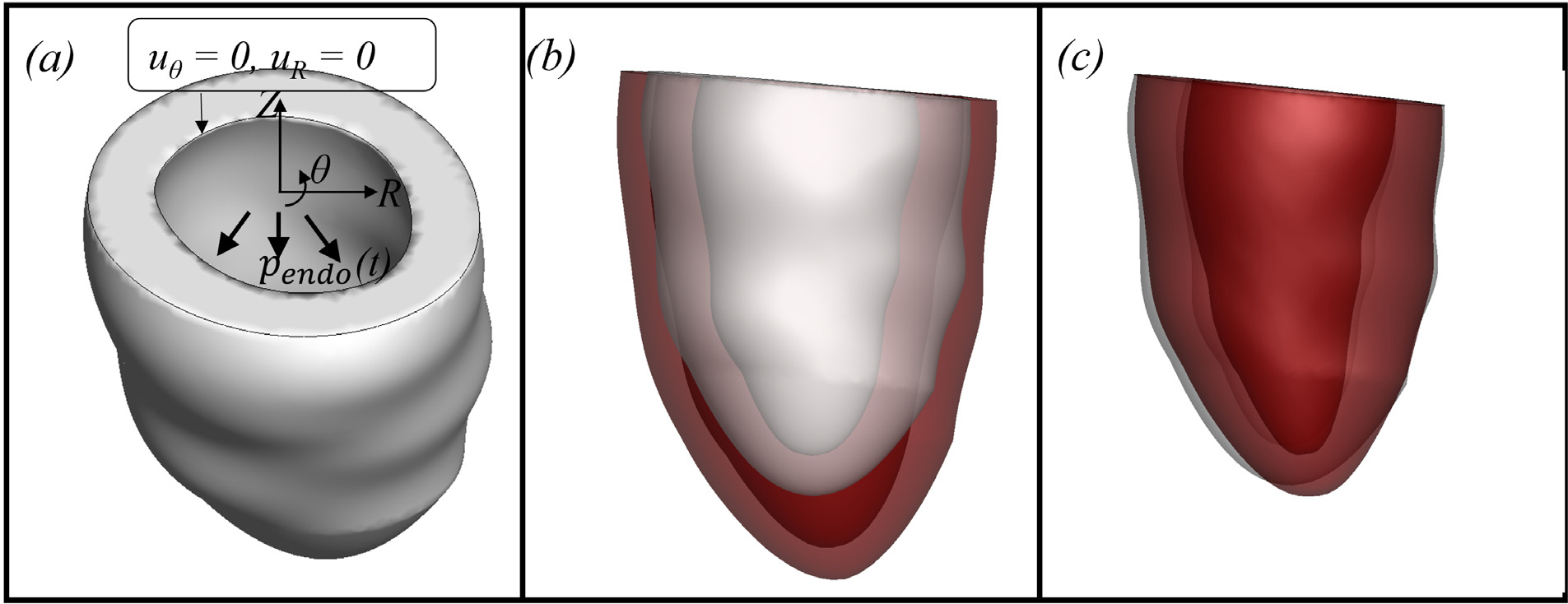
(a) Computational setup for the LV problem and the deformed position of the LV at the (b) end of diastole and (c) peak of systole (Section 4.3). The red colour shows the deformed position and the grey colour shows the reference position.

This section presents numerical simulations of perfusion in the left ventricle using a previously developed image-based geometry [40] shown in Fig. 20. Like many previous studies [11,13,22], we only consider a portion of the LV. As illustrated in Fig. 20a, we apply zero normal and circumferential displacements at the base, allowing only radial expansion along the plane of the base of the LV.

The blood pressure inside the LV is modelled by a uniform pressure throughout the endocardial surface with a prescribed waveform *p*^endo^(*t*). The HO model (Eq. (38)) is used for the hyperelastic strain energy function with solid and fluid densities $\rho_{0}^{\text{s}}=\rho_{0}^{\text{f}}=1.0\;\;\text{g}/{\text{cm}}^{3}$, material constants *a* = 2244.87 dyne*/*cm^2^, *b* = 1.6215, *a*_f_ = 24267 dyne*/*cm^2^, *b*_f_ = 1.8268, *a*_s_ = 5562.38 dyne*/*cm^2^, *b*_s_ = 0.7746, *a*_fs_ = 3905.16 dyne*/*cm^2^, and *b*_fs_ = 1.695, along with initial porosity *ϕ*_0_ = 0.1, permeability **K** = 10^−8^**I** cm^4^dyne^−1^s^−1^, and pore pressure constants *q*_1_ = 220 dyne*/*cm^2^, *q*_2_ = 10090 dyne*/*cm^2^, and *q*_3_ = 75. The anisotropic properties in the myocardial skeleton are modelled using myofibres and collagen sheets as specified in Richardson et al. [13]. The myocardium is considered porous, with source/sink terms dependent on the pore pressure [11],

(78) $$S(X,t)=\beta^{\text{so}\;}\left(p^{\text{so}\;}-p^{\text{pore}\;}(X,t)\right)-\beta^{\text{si}\;}\left(p^{\text{pore}\;}(X,t)-p^{\text{si}\;}\right),$$

in which *β*^so^ and *β*^si^ are constants and *p*^so^ and *p*^si^ are the source and sink pressures. For the numerical simulation, *β*^so^ = *β*^si^ = 3 × 10^−6^ cm^2^dyne^−1^s^−1^, *p*^so^ = 2.7 × 10^4^ dyne*/*cm^2^, and *p*^si^ = 1.3 × 10^4^ dyne*/*cm^2^ are used.

We simulate three consecutive cardiac cycles, including diastolic and systolic phases. The endocardial pressure is applied with a maximum values of 1.067 kPa and 14.53 kPa during the diastole and systole, respectively, given by

(79) $$p^{\text{endo}}(t)=\{\begin{array}{ll} 1.067\frac{t}{0.2}\;\text{kPa}, & \;\text{if}\;t<0.2,\\ 1.067\;\text{kPa}, & \;\text{if}\;0.2<t<0.5,\\ 1.067+13.46\left(1-\exp\left(-\frac{{\left(t-0.5\right)}^{2}}{0.004}\right)\right)\;\text{kPa}, & \;\text{if}\;0.5<t<0.65,\\ 1.067+13.46\left(1-\exp\left(-\frac{{\left(0.8-t\right)}^{2}}{0.004}\right)\right)\text{kPa}, & \;\text{if}\;0.65<t<0.8. \end{array}$$

For computing the active component of the stress tensor (Eq. (40)), the active tension (Eq. (41)) is prescribed using the maximum values of active tension from a healthy LV model [41], given as

(80) $$T_{\text{a}}(t)=\{\begin{array}{ll} 0\;\text{kPa}, & \;\text{if}\;t<0.5,\\ 84.26\left(1-\exp\left(\frac{-{\left(t-0.5\right)}^{2}}{0.005}\right)\right)\;\text{kPa}, & \;\text{if}\;0.5<t<0.65,\\ 84.26\left(1-\exp\left(\frac{-{\left(0.8-t\right)}^{2}}{0.005}\right)\right)\;\text{kPa}, & \;\text{if}\;0.65<t<0.8. \end{array}$$

**Fig. 21.**
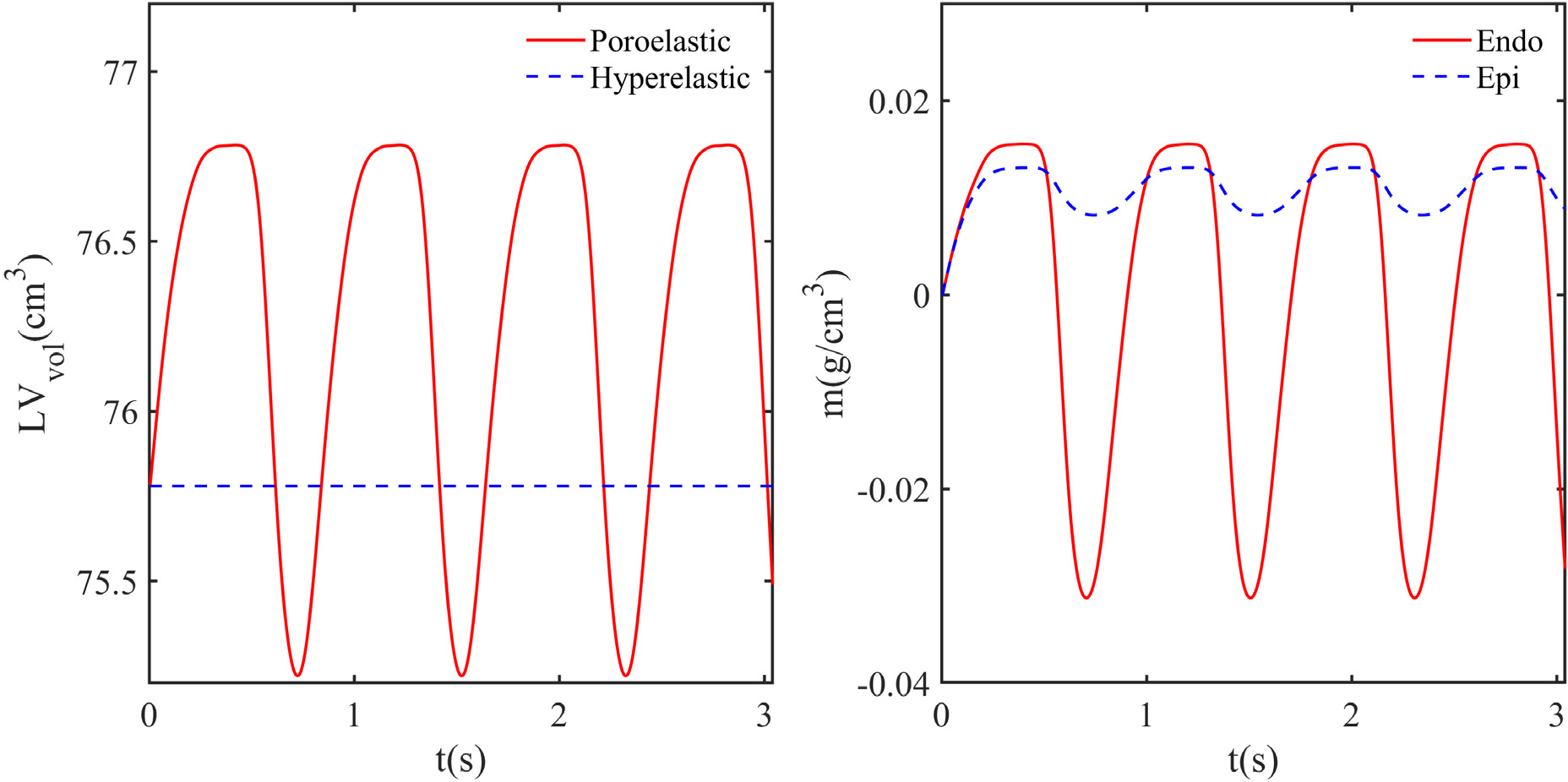
Temporal variation of the volume of the myocardium *LV*_vol_ for the poroelastic and hyperelastic cases and added mass *m* at the epicardial and endocardial surfaces, for three complete cardiac cycles.

Fig. 20b and c show the deformed positions at the end of diastole and systole, respectively. Fig. 21a shows the temporal variation of the volume of the myocardium for both hyperelastic and poroelastic cases for three complete cycles. The hyperelastic simulations shown here are carried out using the same methodology but with zero porosity and external source. For both cases, a periodic state is obtained by the third cycle. The volume remains almost constant throughout the cardiac cycle for the hyperelastic case because the myocardium is treated as incompressible. For the poroelastic case, the volume changes depending on the added mass. Note that the volume of the skeleton $\left(J-m/\rho_{0}^{\text{f}}\right)$ remains almost constant throughout the cycle.

**Fig. 22.**
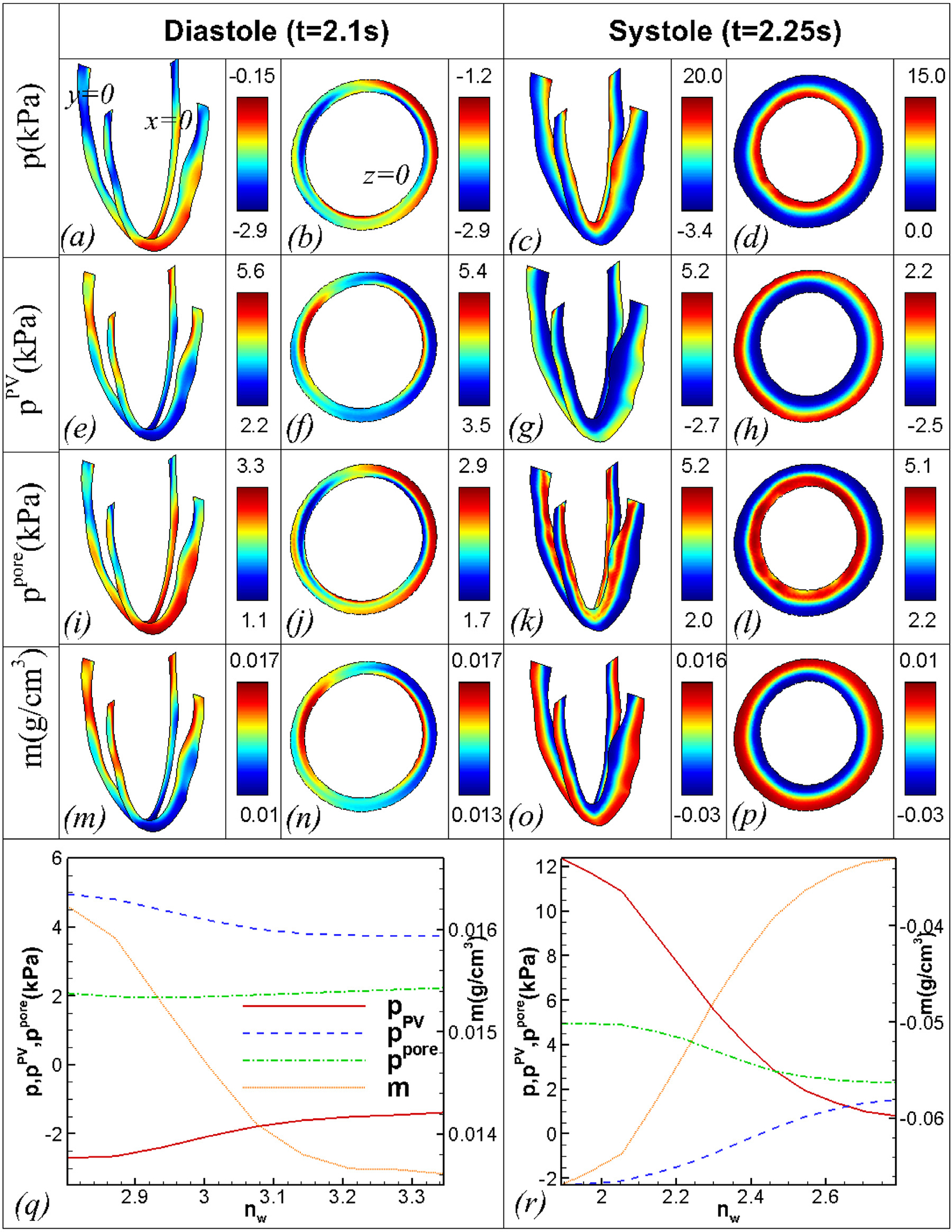
Fields of (a) - (d) *p*, (e) - (h) *p*^PV^, (i) - (l) *p*^pore^, and (m) - (p) *m* at different cross sections of the LV (*X* = 0cm, *Y* = 0cm, and *Z* = 3.0cm) at the end of the diastole *t* = 2.1s and the peak of systole *t* = 2.25s, and the variation of *p*, *p*^PV^, *p*^pore^, and *m* across the line marked in the plane *Z* = 3.0cm, at the (q) end of diastole and (r) the peak of systole.

In our poroelastic formulation, the change in the added mass *m* is governed by the Darcy flow pore fluid model, and the fluid flow into or out of the pores depends on the pore pressure-dependent source/sink. Fig. 21b shows the temporal variation of the space-averaged *m* for the endocardial and epicardial surfaces. During the diastolic phase, *m* increases on both surfaces because of the smaller pore pressure, which results in fluid flow from the source to the pores. Notice that at the end of diastole, the average *m* is larger at the endocardium than the epicardium. Furthermore, *m* falls at both the endocardium and epicardium during the systole. However, the rate of decrease in *m* is greater near the endocardium. The variation of *m* at the epicardium and endocardium in the diastole and systole could be attributed to the larger pore pressure at the epicardium (endocardium) during the diastolic (systolic) phase (Fig. 22). Fig. 22 shows the fields of *p*, *p*^PV^, and *p*^pore^ at various cross-sections of the LV at the periodic state. Fig. 22a, b, and q show that, the pressure is negative everywhere at the end of the diastole. This is because the inflation elongates the fibres that are tangential to the surfaces at the epicardium and endocardium. Thus, the tensile forces along the fibres make the volumetric component of the total fibre stress and hence the pressure negative [8,42,43]. Further, the magnitude of the negative pressure is larger near the endocardium than the epicardium. This results from larger fibre elongation and the associated fibre stress near the endocardium as compared to the epicardium (Fig. 22q). In addition, the pressure is larger near the base than the middle and apex portions. This can be attributed to the constraints imposed on the displacements of the base and associated reductions in the fibre stretch and fibre stress. The fibre stretch and the fibre stress are also smaller near the apex than the middle portion because of the restricted motion near the apex, resulting in a slightly larger pressure near the apex. Fig. 22b shows that the pressure variation in the transmural direction is different at different angular locations because of the asymmetric deformation. During the systolic phase, Fig. 22c, d, and r show that *p* achieves a maximum near the endocardium and a minimum near the epicardium. Further, the pressure is positive everywhere because the fibres are compressed during contraction, and the fibre stress is positive. The fibre stress is more near the endocardium than the epicardium (Fig. 22e). Thus, the compressive force and associated pressure are greater near the endocardium than the epicardium.

**Fig. 23.**
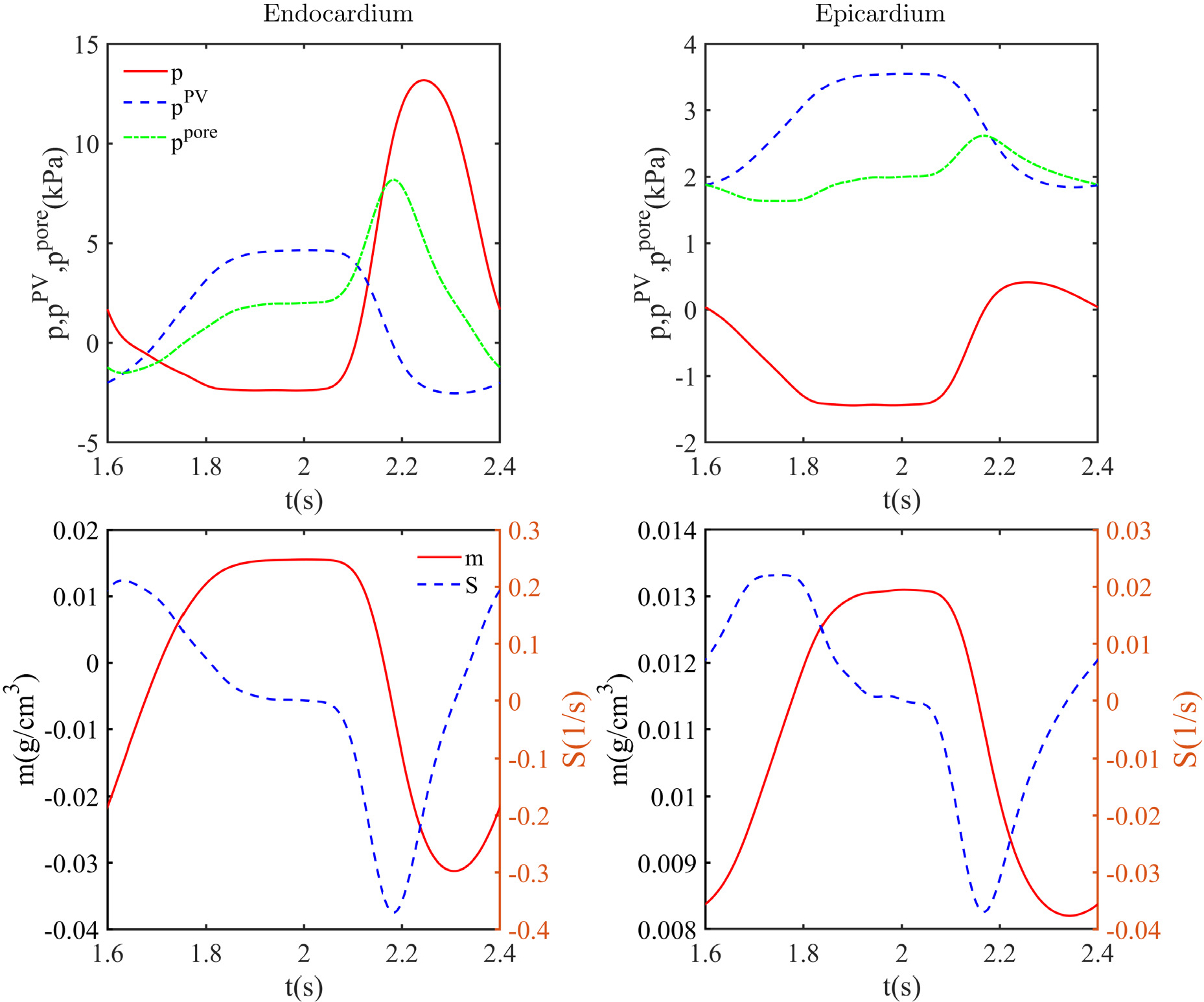
Temporal variation of *p*, *p*^PV^, *p*^pore^, *m*, and *S* at the endocardial and epicardial surfaces during the third cardiac cycle.

Fig. 23 shows temporal variations of the pore pressure *p*^pore^, its components *p* and *p*^PV^, the added mass *m*, and the source *S*. Note that when *m* = 0, *p*^PV^ is zero. Thus, *p* is the pressure driving the source *S* and the added mass *m* in the beginning of the cycle. Because of the negative pressure during the diastole (Fig. 22b), the associated smaller pore pressure results in fluid flow from the source to the pores and an increase in the added mass *m*. With increasing *m*, *p*^PV^ increases exponentially, leading to a positive total pore pressure *p*^pore^ at both surfaces (Fig. 22j). However, the total pore pressure *p*^pore^ is less than the source pressure of 2.7 kPa, so that there is net flow into the myocardium. Thus, the added mass *m* increases at the epicardium and the endocardium throughout the diastole (Fig. 23). Note that, the space averaged *p* and the pore pressure *p*^pore^ are slightly larger near the epicardium than the endocardium at the end of the diastole (Fig. 22q). Thus, the added mass *m* is slightly larger near the endocardium than the epicardium. By the end of the diastole, the total pore pressure *p*^pore^ nearly balances with the source and sink pressures, and the source *S* is nearly zero (Fig. 23), leading to a constant added mass *m*.

Fig. 23 shows that *p* increases rapidly at both the endocardium and the epicardium during systole. The pressure *p* is very large near the endocardium as compared to the epicardium (Fig. 22r). At both surfaces, *p*^pore^ increases and the associated source *S* rapidly decreases (Fig. 23), leading to the shrinking of the pores, similar to that reported in the literature [11,22,42]. This leads to a rapid decrease in the added mass *m*. Note that the rate of decrease of *m* is very large near the endocardium as compared to the epicardium. This is because the pore pressure *p*^pore^ is larger near the endocardium than the epicardium (Fig. 22r). Since *p*^PV^ also increases exponentially with increasing *m* (Fig. 23), the rate of increase of *p*^pore^ is less than that of *p*. Furthermore, the source *S* reaches its minimum value for both the endocardium and the epicardium when the pore-fluid pressure *p*^pore^ reaches its maximum (Fig. 23). However, the added mass *m* continues to drop until the source *S* becomes positive (Fig. 23). We remark that the order of magnitude and the type of variation of the pore pressure are in good agreement with the literature [11,22].

## 5. Conclusion

This paper develops a stabilized equal-order mixed finite element method for anisotropic incompressible hyperelastodynamics and poroelastodynamics using linear finite elements. Our approach extends a dynamic variational multiscale method, presented by Rossi et al. [33] for incompressible isotropic hyperelastodynamics, to anisotropic hyperelastodynamics and poroelastodynamics. We verify the method’s convergence, stability, and robustness for various anisotropic hyperelastic problems and isotropic/anisotropic poroelastic problems. We find that our method results in second-order accuracy in space and time, and the results are in excellent agreement with the standard selective reduced integration method (*Q*^1^–*B*-bar). For poroelastodynamics, our results are in excellent agreement with the benchmark results of Chapelle et al. [11]. In addition, poroelastic simulations of ventricular perfusion are performed using a realistic left ventricular geometry. Good qualitative agreement is obtained with prior results reported in the literature. The temporal variation of the various components of the pore pressure are correlated with the variation of the added mass *m* and the dynamics of the myocardium. The anisotropic behaviour greatly influences the magnitude of the pore pressure and its distribution across the myocardium. The skeleton pressure during the diastole (systole) was found to be negative (positive) in most of the places due to tension (compression) on the myofibres.

## Data Availability

Data will be made available on request.

## Appendix A. Analytical solution of hyperelastic shear deformation

For the shear deformation described by Eq. (70), the deformation gradient tensor **F** is

(A.1)
$$F(X,t)=\left[\begin{array}{ccc} 1 & 0 & 2\alpha Z\sin(t)\\ 0 & 1 & \beta \sin\;(t)\\ 0 & 0 & 1 \end{array}\right].$$

Note that det(**F**) = 1 satisfies the mass balance condition. The right Cauchy–Green strain is

(A.2)
$$C(X,t)=\left[\begin{array}{ccc} 1 & 0 & 2\alpha Z\sin\;(t)\\ 0 & 1 & \beta \sin\;(t)\\ 2\alpha Z\sin\;(t) & \beta \sin\;(t) & -4\alpha^{2}\cos^{2}\;(t)Z^{2}+4\alpha^{2}Z^{2}-\beta^{2}\cos^{2}\;(t)+\beta^{2}+1 \end{array}\right].$$

The first invariant of **C** is

(A.3)
$$I_{1}=3-4\alpha^{2}{\text{cos}}^{2}\;(t)Z^{2}+4\alpha^{2}Z^{2}-\beta^{2}{\text{cos}}^{2}\;(t)+\beta^{2}.$$

Using the initial fibre direction $f_{0}=\frac{1}{\sqrt{3}}(1,1,1)$, the fibre invariant is

(A.4)
$$I_{4\text{f}}=\frac{\left(-4\alpha^{2}Z^{2}\;-\;\beta^{2}\right)\cos^{2}\;(t)}{3}+\frac{\left(4\alpha Z+2\beta )\sin\;(t\right)}{3}\;+\;\frac{4\alpha^{2}Z^{2}}{3}+\frac{\beta^{2}}{3}+1.$$

The deviatoric part of the first Piola–Kirchhoff stress tensor is

(A.5)
$$\mathrm{Dev}[P]=G\left(F-\frac{I_{1}}{3}\cdot F^{-T}\right)+2G_{\text{f}}\left(Ff_{0}⊗f_{0}-\frac{I_{4\text{f}}}{3}\cdot F^{-T}\right).$$

The inertia term in the momentum equation is

(A.6)
$$\rho_{0}^{\text{f}}v=\left(-\rho_{0}^{\text{f}}\alpha Z^{2}\omega^{2}\sin\;(t)\right)e_{X}+\left(-\rho_{0}^{\text{f}}\beta Z\omega^{2}\sin\;(t)\right)e_{Y}+(0)e_{Z}.$$

Using momentum balance, the pressure can be shown to satisfy

(A.7)
$$\nabla_{X}\cdot (pH)=-\rho_{0}^{\text{f}}I+\nabla_{X}\cdot (\mathrm{Dev}[P])+\rho_{0}^{\text{f}}b.$$

With the body force

(A.8)
$$b=(0)\;e_{X}+(0)\;e_{Y}+\left(\sin(t)\omega^{2}(2\alpha XZ+\beta Y)\right)\;e_{Z},$$

we have

(A.9a)
$$\frac{\partial p}{\partial X}=\frac{\alpha \sin(t)\left(3\rho_{0}^{\text{f}}Z^{2}\;+\;6G\;+\;4G_{\text{f}}\right)}{3},$$

(A.9b)
$$\frac{\partial p}{\partial Y}=\rho_{0}^{\text{f}}\beta Z\sin(t),$$

(A.9c)
$$\begin{array}{l} \frac{\partial p}{\partial Z}=-\frac{8}{3}\left(\frac{3}{4}\alpha Z\left(\frac{\partial p}{\partial X}\right)+\frac{3}{8}\beta \left(\frac{\partial p}{\partial Y}\right)+\alpha^{2}Z\left(G+\frac{2G_{\text{f}}}{3}\right)\sin\;(t)\right)\sin\;(t)\\ \;+\left(\left(-\frac{3\rho_{0}^{\text{f}}ZX}{4}+\frac{G_{\text{f}}}{3}\right)\alpha -\frac{3\rho_{0}^{\text{f}}\beta Y}{8}\right)\sin\;(t). \end{array}$$

The above system of partial differential equations are solved to obtain

(A.10)
$$\begin{array}{l} \;p=-\frac{10}{3}\left(\left(\frac{3\rho_{0}^{\text{f}}Z^{2}}{20}+G+\frac{2G_{\text{f}}}{3}\right)\alpha^{2}+\frac{3\beta^{2}\rho_{0}^{\text{f}}}{20}\right)Z^{2}\sin^{2}\;(t)+\\ \frac{1}{18}\left(\left(18XZ^{2}\rho_{0}^{\text{f}}-16ZG_{\text{f}}+36\left(G+\frac{2G_{\text{f}}}{3}\right)X\right)\alpha +18\rho_{0}^{\text{f}}\beta ZY\right)\sin\;(t). \end{array}$$

## Appendix B. Analytical solution for poroelastic shear deformation

For the poroelastic shear deformation described by Eq. (75), the deformation gradient is

(B.1)
$$F(X,t)=\left[\begin{array}{ccc} 1+\alpha Z\sin\;(t) & 0 & \alpha X\sin\;(t)\\ 0 & 1 & \beta \sin\;(t)\\ 0 & 0 & 1 \end{array}\right].$$

From $J-1-m/\rho_{0}^{f}=0$, the added mass *m* is

(B.2)
$$m(X,t)=\rho_{0}^{\text{f}}\alpha Z\sin(t).$$

The right Cauchy–Green strain **C** is

(B.3)
$$C(X,t)=\left[\begin{array}{ccc} \left(1+\alpha Z\;\sin^{2}\;(t)\right) & 0 & \left(1+\alpha Z\sin\;(t))\alpha X\sin\;(t\right)\\ 0 & 1 & \beta \sin(t)\\ \left(1+\alpha Z\sin\;(t))\alpha X\sin\;(t\right) & \beta \sin\;(t) & -\alpha^{2}\cos^{2}\;(t)X^{2}+\alpha^{2}X^{2}-\beta^{2}\cos^{2}\;(t)+\beta^{2}+1 \end{array}\right],$$

and the first invariant of **C** is

(B.4)
$$I_{1}=-\alpha^{2}\cos^{2}\;(t)X^{2}-\alpha^{2}\cos^{2}\;(t)Z^{2}+\alpha^{2}X^{2}+\alpha^{2}Z^{2}-\beta^{2}\cos^{2}\;(t)+2\alpha Z\sin\;(t)+\beta^{2}+3.$$

Using the neo-Hookean model, the divergence of the deviatoric part of PK1 stress is given as

(B.5)
$$\begin{array}{l} \;\nabla_{X}\cdot (\mathrm{Dev}[P])=\left(-\frac{4G\alpha^{2}X\sin^{2}\;(t)}{3{\left(1\;+\;\alpha Z\sin\;(t)\right)}^{\frac{5}{3}}}\right)e_{X}+\left(-\frac{2G\beta \sin^{2}\;(t)\alpha }{3{\left(1\;+\;\alpha Z\sin\;(t)\right)}^{\frac{5}{3}}}\right)e_{Y}\\ +\frac{{\left(1\;+\;\alpha Z\sin\;(t)\right)}^{\frac{-5}{3}}}{9}(-\sin\;(t)G\alpha (11\alpha^{2}\cos^{2}\;(t)X^{2}-\alpha^{2}\cos^{2}\;(t)Z^{2}\;+\;5\beta^{2}\cos^{2}\;(t)\\ \;-11\alpha^{2}X^{2}\;+\;\alpha^{2}Z^{2}\;+\;2\alpha Z\sin\;(t)-5\beta^{2}-3))e_{Z}. \end{array}$$

The inertia term is

(B.6)
$$\rho_{0}^{\text{f}}\dot{v}=\left(-\rho_{0}^{\text{f}}\alpha XZ\sin\;(t)\right)\;e_{X}+\left(-\rho_{0}^{\text{f}}\beta Z\sin\;(t)\right)\;e_{Y}+(0)\;e_{Z}.$$

Using the following expression for the body force,

(B.7)
$$\begin{array}{r} \;b=(0)\;e_{X}+(0)\;e_{Y}+\\ (\frac{\sin\;(t)}{18(1\;+\;\alpha Z\sin\;(t))}\left(-9\left(Z^{2}\alpha \left(\alpha^{2}X^{2}+2\beta^{2}\right)\sin^{2}\;(t)+2\sin\;(t)\beta^{2}Z-\alpha X^{2}-2\beta Y\right)\rho_{0}^{\text{f}}\right)+\\ \;\frac{22\sin\;(t)\left(\sin^{2}\;(t)G\left(Z\alpha \left(\alpha^{2}X^{2}\;+\;\frac{3\beta^{2}}{11}\right)\sin\;(t)\;+\;\alpha^{2}X^{2}\;+\;\frac{8\alpha \beta Y}{11}\;+\;\frac{3\beta^{2}}{11}\right)\alpha \right)}{9{\left(1\;+\;\alpha Z\sin\;(t)\right)}^{\frac{8}{3}}}+\\ \;\rho_{0}^{\text{f}}\dot{v}-\nabla_{X}\cdot (\mathrm{Dev}[P]))\;e_{Z}, \end{array}$$

momentum balance implies

(B.8a)
$$\frac{\partial p}{\partial X}=\frac{X\left(\rho_{0}^{\text{f}}Z{\left(1\;+\;\alpha Z\sin\;(t)\right)}^{\frac{5}{3}}\;-\;\frac{4G\alpha \sin(t)}{3}\right)\sin\;(t)\alpha }{{\left(1\;+\;\alpha Z\sin\;(t)\right)}^{\frac{5}{3}}},$$

(B.8b)
$$(1\;+\;\alpha Z\sin\;(t))\frac{\partial p}{\partial Y}=\frac{\left(\rho_{0}^{\text{f}}Z{\left(1\;+\;\alpha Z\sin\;(t)\right)}^{\frac{5}{3}}\;-\;\frac{2G\alpha \sin(t)}{3}\right)\sin\;(t)\beta }{{\left(1\;+\;\alpha Z\sin\;(t)\right)}^{\frac{5}{3}}},$$

(B.8c)
$$\begin{array}{l} \;-\;\alpha X\sin\;(t)\frac{\partial p}{\partial X}\;-\;(1\;+\;\alpha Z\sin\;(t))\;\beta \sin\;(t)\frac{\partial p}{\partial Y}\;+\;(1\;+\;\alpha Z\sin\;(t))\frac{\partial p}{\partial Z}=\\ \;-\;\frac{\sin\;(t)G\alpha }{9{\left(1\;+\;\alpha Z\sin\;(t)\right)}^{\frac{5}{3}}}(11\alpha^{2}\cos^{2}\;(t)X^{2}\;-\;\alpha^{2}\cos^{2}\;(t)Z^{2}\;+\;5\beta^{2}\cos^{2}\;(t)\;-\;11\alpha^{2}X^{2}\;+\;\\ \alpha^{2}Z^{2}\;+\;2\alpha Z\sin\;(t)\;-\;5\beta^{2}\;-\;3)\;+\;b_{Z}. \end{array}$$

The above system of equations can be solved to obtain

(B.9)
$$\begin{array}{l} p=\;-\;\frac{2X^{2}\left(\;-\;\frac{3\rho_{0}^{\text{f}}Z{\left(1+\alpha Z\sin(t)\right)}^{\frac{5}{3}}}{4}\;+\;G\alpha \sin\;(t)\right)\sin\;(t)\alpha }{3{\left(1\;+\;\alpha Z\sin\;(t)\right)}^{\frac{5}{3}}}\;+\;\\ \;\frac{\left(\rho_{0}^{\text{f}}Z{\left(1\;+\;\alpha Z\sin\;(t)\right)}^{\frac{5}{3}}\;-\;\frac{2G\alpha \sin(t)}{3}\right)\sin\;(t)\beta Y}{{\left(1\;+\;\alpha Z\sin\;(t)\right)}^{\frac{8}{3}}}. \end{array}$$

The pore pressure *p*^pore^ is

(B.10)
$$\begin{array}{l} \;p^{\text{pore}\;}=\;-\;\frac{2X^{2}\left(\;-\;\frac{3\rho_{0}^{\text{f}}Z{\left(1+\alpha Z\sin(t)\right)}^{\frac{5}{3}}}{4}\;+\;G\alpha \sin\;(t)\right)\sin\;(t)\alpha }{3{\left(1\;+\;\alpha Z\sin\;(t)\right)}^{\frac{5}{3}}}\;+\;\\ \frac{\left(\rho_{0}^{\text{f}}Z{\left(1\;+\;\alpha Z\sin\;(t)\right)}^{\frac{5}{3}}\;-\;\frac{2G\alpha \sin(t)}{3}\right)\sin\;(t)\beta Y}{{\left(1\;+\;\alpha Z\sin\;(t)\right)}^{\frac{8}{3}}}\;+\;\kappa \rho_{0}^{\text{f}}\alpha Z\sin\;(t). \end{array}$$
